# ASCL1 activates neuronal stem cell-like lineage programming through remodeling of the chromatin landscape in prostate cancer

**DOI:** 10.1038/s41467-022-29963-5

**Published:** 2022-04-27

**Authors:** Shaghayegh Nouruzi, Dwaipayan Ganguli, Nakisa Tabrizian, Maxim Kobelev, Olena Sivak, Takeshi Namekawa, Daksh Thaper, Sylvan C. Baca, Matthew L. Freedman, Adeleke Aguda, Alastair Davies, Amina Zoubeidi

**Affiliations:** 1 Department of Urologic Sciences, University of British Columbia, Vancouver, BC V5Z 1M9 Canada; 2 Vancouver Prostate Centre, Vancouver, BC V6H 3Z6 Canada; 3 Departement of Medical Oncology, Dana-Farber Cancer Institute, Boston, MA United States

**Keywords:** Prostate cancer, Epigenetics, Gene expression

## Abstract

Treatment with androgen receptor pathway inhibitors (ARPIs) in prostate cancer leads to the emergence of resistant tumors characterized by lineage plasticity and differentiation toward neuroendocrine lineage. Here, we find that ARPIs induce a rapid epigenetic alteration mediated by large-scale chromatin remodeling to support activation of stem/neuronal transcriptional programs. We identify the proneuronal transcription factor ASCL1 motif to be enriched in hyper-accessible regions. ASCL1 acts as a driver of the lineage plastic, neuronal transcriptional program to support treatment resistance and neuroendocrine phenotype. Targeting ASCL1 switches the neuroendocrine lineage back to the luminal epithelial state. This effect is modulated by disruption of the polycomb repressive complex-2 through UHRF1/AMPK axis and change the chromatin architecture in favor of luminal phenotype. Our study provides insights into the epigenetic alterations induced by ARPIs, governed by ASCL1, provides a proof of principle of targeting ASCL1 to reverse neuroendocrine phenotype, support luminal conversion and re-addiction to ARPIs.

## Abstract (web summary)

Following androgen receptor pathway inhibition prostate cancers can differentiate towards the neuroendocrine lineage. Here, the authors identify epigenetic alterations regulated by ASCL1 and suggest targeting ASCL1 to reverse the neuroendocrine phenotype.

## Introduction

Potent androgen receptor (AR) pathway inhibitors (ARPIs), such as enzalutamide (ENZ) and abiraterone (Abi), have increased patient survival with advanced prostate cancer disease;^1,2^ however, resistance ultimately occurs. In particular, a subset of tumors shed their luminal identity and dependency on the canonical AR signaling. These variants exhibit lineage plasticity and neuroendocrine differentiation^3–8^ and are referred to as treatment-induced neuroendocrine prostate cancer (t-NEPC). While de novo NEPC is rare^9,10^, development of treatment-induced NEPC accounts for 20% of advance, treatment-refractory castration-resistant prostate cancer (CRPC)^3,5,11^. NEPC is characterized by loss of canonical AR signaling and expression of neuronal lineage markers, such as chromogranin (CHGA) and synaptophysin (SYP), distinct small cell morphology^4,7,12^, along with a stem cell transcriptional program^13^. Apart from alterations in RB1 and TP53, which have been associated with lineage plastic phenotypes^14^, CRPC and NEPC share relatively similar genomic landscape^15,16^. The evolution of CRPC to NEPC is accompanied by extensive transcriptional reprogramming^12,15^, suggesting that the emergence of a neuroendocrine phenotype may be driven predominantly by epigenetic dysregulation. The heterogeneous nature of prostate cancer^17,18^ provides possibility of multiple drivers for the transition of the lineage under the pressure of current therapeutic strategies. It is still unclear, mechanistically, how tumors govern variation in response to treatment and how they define alternative cell fate post ARPI.

In this work, we investigate the epigenetic landscape of CRPC after ENZ treatment by profiling global chromatin accessibility to uncover the earliest factors that drive cellular plasticity and commitment to the neuroendocrine lineage. We find that the DNA binding motif for the neuronal lineage-guiding transcription factor ASCL1 becomes hyper-accessible following ENZ treatment and ASCL1 is required for ENZ-induced lineage plasticity. Loss of ASCL1 expression alters the epigenetic programming in t-NEPC by disrupting the polycomb repressive complex 2 (PRC2) and reducing the EZH2 chromatin bound to support the lineage reversal to a luminal AR-driven state. This effect is attributed to an increase of p-EZH2-T311 through UHRF1/AMPK axis.

## Results

### An epigenetic plasticity emerges in response to hormone therapy

**Fig. 1:**
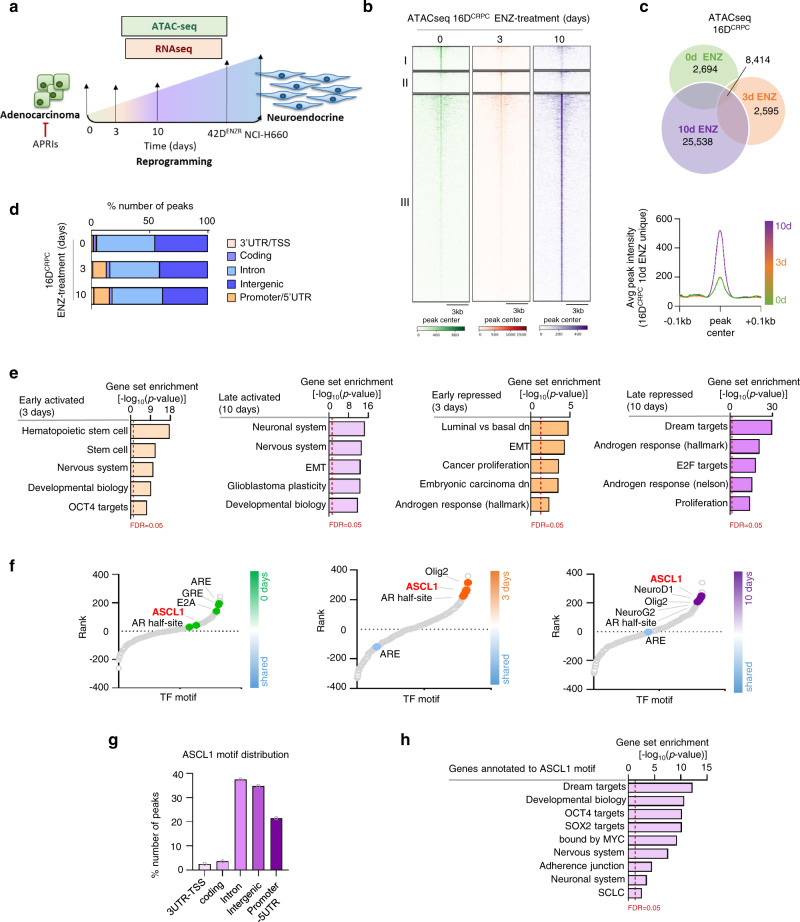
Hormone therapy triggers epigenetic plasticity. **a** Schematic representation of the study outline. **b** Heatmap indicating accessibility in 16D^CRPC^ with enzalutamide (ENZ) treatment for 0, 3, or 10 days (*n* = 3 biologically independent samples). **c** Number of ATACseq peak (top) average signal profile from unique 16D^CRPC^ 10 day ENZ-treated regions (bottom). **d** Genomic annotation of all accessible peaks presented as percentage of all peaks. **e** Gene set enrichment analysis (GSEA) shows transcriptional response to ENZ treatment associated with gain/loss accessibility in 16D^CRPC^ cells represented as: early repressed; late repressed; early activated; late activated. Dotted line represent false discovery rate (FDR) of 0.05, *p* < 0.05, statistical analysis was performed using a hypergeometric test. See also Supplementary Fig. 1. **f** Transcription factor (TF) binding motifs surrounding accessible chromatin in unique vs. shared regions, ranked based on differential *p*-value. Each dot represents a motif. Statistical analysis was performed using a cumulative hypergeometric test. **g** Genomic annotation for genes mapped to ASCL1 motif in 16D^CRPC^ 10 days ENZ-treated, presented as percentage of all peaks. See also Supplementary Fig. 1. **h** Pathways associated to genes mapped to ASCL1 motif in 16D^CRPC^ 10 days ENZ-treated. Dotted line represents FDR = 0.05, *p* < 0.05, statistical analysis was performed using a hypergeometric test. See also Supplementary Fig. 1. Source data are provided as a Source Data file.

Despite similar genetic profiles, the conversion from CRPC to NEPC post ARPIs is accompanied by extensive transcriptional re-wiring^12,15^. This suggests that reprogramming of the chromatin landscape may play a central role in this lineage plasticity. To explore this premise, we interrogated changes in the chromatin landscape of CRPC cells post ENZ treatment by performing assay for transposase-accessible chromatin using sequencing (ATACseq) (Fig. 1a). We observed that acute ENZ treatment (3 days) led to altered chromatin accessibility with 2,595 regions (Fig. 1b, region II) gaining accessibility at 3 days post-treatment compared to 10 days and non-treated CRPC. By 10 days post-treatment, widespread changes in chromatin accessibility were observed with 25,538 newly hyper-accessible regions (Fig. 1b, region III) when compared to 3 days and non-treated CRPC, while 2,694 accessible regions (Fig. 1b, region I) were accessible in non-treated CRPC only (Fig. 1b, c). A slight bias toward increased accessibility of promoter regions following treatment of ENZ was observed (Fig. 1d). To further investigate the significance of regions affected by ENZ treatment (opened or closed specifically in response to ENZ), we integrated ATACseq with RNAseq from matched treatment. We defined activated as newly accessible and expressed genes (described as 50 kb distance to accessible peak center with expression of log2Fold Change>1, <-1) in response to ENZ. While, genes that lost accessibility and expression following ENZ treatment were defined as repressed. We found that ENZ redirected the chromatin accessibility from canonical “AR-driven” transcriptional program in CRPC to positively regulated pathways involved in cell plasticity. Importantly, we observed that pathways involved in stem cells were highly enriched compared to the neuronal pathways at 3 days while at 10 days post-treatment, the neuronal pathways become more enriched. As expected, repression of canonical AR transcriptional program was observed at 3 days, with further repression at 10 days post-treatment (Fig. 1e and Supplementary Fig. 1a, b).

To identify potential regulators of this large-scale epigenetic reprogramming in response to ARPI, we performed transcription factor (TF) motif analysis within a 50 bp window surrounding ATACseq peaks and discovered that both androgen-response element and glucocorticoid response element are the most enriched motifs in 16D^CRPC^, while consensus binding sequence for neuronal lineage TFs (ASCL1, Olig2, NeuroD1, and NeuroG2) were enriched in ENZ-treated CRPC (Fig. 1f). Ranking motifs by p-value, we found the DNA binding motif for the pro-neural TF ASCL1 to be disproportionally enriched in hyper-accessible chromatin regions post ENZ treatment (Fig. 1f, Supplementary Data 2). Specifically, ASCL1 was the most favorable TF, as their rank went from 126^th^ place in non-treated cells to 8^th^ after 3 days and 4^th^ after 10 days of ENZ treatment. ASCL1 motif is highly accessible in NE-like state GEM model^14^ compared to adenocarcinoma (Supplementary Fig. 1c). Analysis of genomic distribution of ASCL1 motif in unique accessible regions of 10 days ENZ-treated CRPC (Supplementary Fig. 1d and Supplementary Data 3), revealed a bias towards enhancer regions (intronic and intergenic) (Fig. 1g and Supplementary Fig. 1e). In particular, regions associated with DNA-binding motif of ASCL1 were enriched for genes associated with both stem and neuronal lineage programing (Fig. 1h and Supplementary Fig. 1f). These data suggest that ENZ induces a well-organized chromatin dynamic that function to unlock lineage plasticity that may support treatment resistance.

### Enzalutamide-mediated chromatin remodeling supports neuroendocrine differentiation

**Fig. 2:**
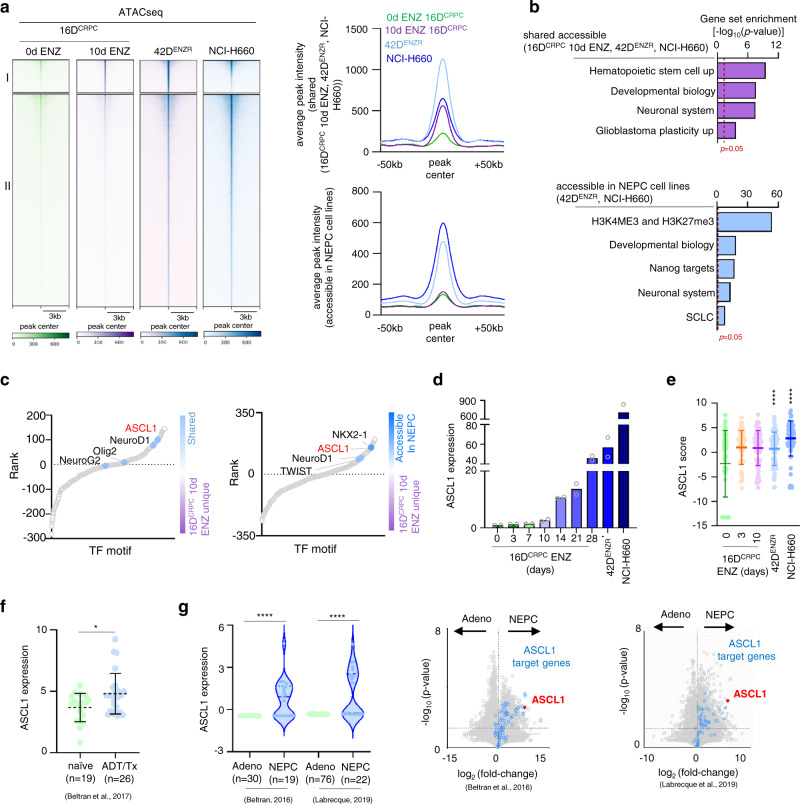
Enzalutamide-induced epigenetic plasticity leads to neuronal differentiation. **a** Heatmap showing chromatin accessibility comparing 16D^CRPC^ 10 days ENZ-treated unique accessible regions to neuroendocrine prostate cancer (NEPC) (left) average signal profile (right). **b** GSEA in accessible shared regions (top) and accessible in NEPC (bottom). Dotted line represent *p* = 0.05, statistical analysis was performed using a hypergeometric test. See also Supplementary Fig. 2. **c** TF binding motifs surrounding accessible chromatin in shared accessible region (left) accessibility in NEPC (right) vs. unique to 16D^CRPC^ 10 days ENZ-treated, ranked based on differential *p*-value. Each dot represents a motif. Statistical analysis was performed using a cumulative hypergeometric test. **d** ASCL1 mRNA expression normalized to GAPDH (*n* = 2 biologically independent samples). **e** ASCL1 score reported as log2FPKM mean ± SD. (16D^CRPC^ *n* = 3, 16D^CRPC^ 3d ENZ *n* = 1, 16D^CRPC^ 10d ENZ *n* = 1, 42D^ENZR^ and NCI-H660 *n* = 3 biologically independent samples. 42D^ENZR^ and NCI-H660 *p* < 0.0001; two-tailed *p*-value). **f** ASCL1 expression presented as normalized read counts in serial sections from naïve and neoadjuvant androgen-deprivation therapy (ADT)/TAX-treated prostate tumors^21^. Each dot represent individual patient. Data shown as mean ± SD, with significance assessed using a two-tailed unpaired t-test, *p* = 0.0135. **g** ASCL1 expression reported as log2TPM in adenocarcinoma (Adeno) and NEPC^12, 15^ cohorts. Violin plots show median (middle solid line), quartiles as dotted lines and interquartile range, each dot represent a patient, with significance assessed using a two-tailed unpaired t-test. (Beltran et al. Adeno *n* = 30 and NEPC *n* = 19; and Labrecque et al. Adeno *n* = 76 and NEPC *n* = 22 patients) (left). Volcano plot shows expression and activity of ASCL1 in Beltran and Labrecque cohorts^12, 15^. Each dot represents a gene, with “ASCL1 signature” (blue) and “ASCL1” (red) highlighted. Dotted line represent *p* = 0.05, statistical analysis was performed using a hypergeometric test (right). Source data are provided as a Source Data file.

In order to delineate whether alterations in chromatin landscape mediated by ENZ treatment support a neuronal phenotype, we compared the chromatin accessibility profile (ATACseq) of ENZ-treated CRPC (10 days) with the t-NEPC model (42D^ENZR^) and de novo NEPC cell line (NCI-H660). We found that a number of genomic loci accessible in ENZ-treated CRPC (10 days) remains accessible in NEPC cell lines; ~75% overlap with 42D^ENZR^ and ~40% with NCI-H660 (region I) (Fig. 2a and Supplementary Fig. 2a). This ENZ-induced chromatin remodeling in CRPC was found to be accessible in NEPC cell lines. For instance, analysis of the genomic loci of neuroendocrine genes *CHGA* and *NCAM1* and stem cell gene *SOX2* revealed accessibility of promoter regions of these genes in CRPC cells after ENZ treatment and in NEPC cell lines (Supplementary Fig. 2b). In addition, we observed a further opening of chromatin in NEPC cell lines, with distinct accessible regions (~28,800 peaks shared between NEPC cell lines) (region II) (Fig. 2a and Supplementary Fig. 2a). Genes associated with stem and neuronal transcriptional network were enriched within 16D^CRPC^ 10 days treated with ENZ, 42D^ENZR^, and NCI-H660 shared regions and NEPC cell lines (Fig. 2b and Supplementary Fig. 2c) with ASCL1 binding motifs enriched surrounding accessible peaks (Fig. 2c and Supplementary Data 4).

In agreement with changes in the chromatin architecture, ASCL1 expression rapidly increased upon ENZ treatment of adenocarcinoma cell lines in a time-dependent manner (Fig. 2d), in LNCaP cell lines following androgen deprivation (Supplementary Fig. 2e), and in patient derived xenografts (PDX) models post castration^19^ (Supplementary Fig. 2f). The expression of ASCL1 was maintained in the NEPC cell lines 42D^ENZR^ and NCI-H660 (Fig. 2d) and was correlated with increase of its binding to neuronal genes (Supplementary Fig. 2g) and transcriptional activity, by using an ASCL1 score^20^ (Fig. 2e). Similar to ENZ treatment, siRNA-mediated silencing of AR in CRPC yielded increased expression of ASCL1 (Supplementary Fig. 2h). Attesting to human relevance, patients treated with 4-6 months neoadjuvant hormone therapy^21^ exhibit high expression of ASCL1 when compared to the treatment naïve group (Fig. 2f), which correlated with high expression of SYP (Supplementary Fig. 2i) supporting our in vitro data that ASCL1 upregulation is an early event following suppression of AR signaling in prostate cancer. Moreover, ASCL1 expression was found to be increased in NEPC patient tumors^12,15^ (Fig. 2g) and in neuroendocrine LuCaP PDX models compared to adenocarcinoma^12^ (Supplementary Fig. 2j). Increased of ASCL1 expression and activity was also observed in a GEM model^14^ following prostate-specific deletion of *RB1* and *TP53* that develop NE-like state from adenocarcinoma (Supplementary Fig. 2k). Based on these results, ASCL1 might be an important factor in establishing the NE lineage identity in prostate cancer. Our results are in alignment with previous studies, showing direct reprograming of induced pluripotent stem cells (iPS) to functional neurons by ectopic ASCL1 expression^22,23^.

### ASCL1 is required to establish the neuronal and stem cell-like lineage

**Fig. 3:**
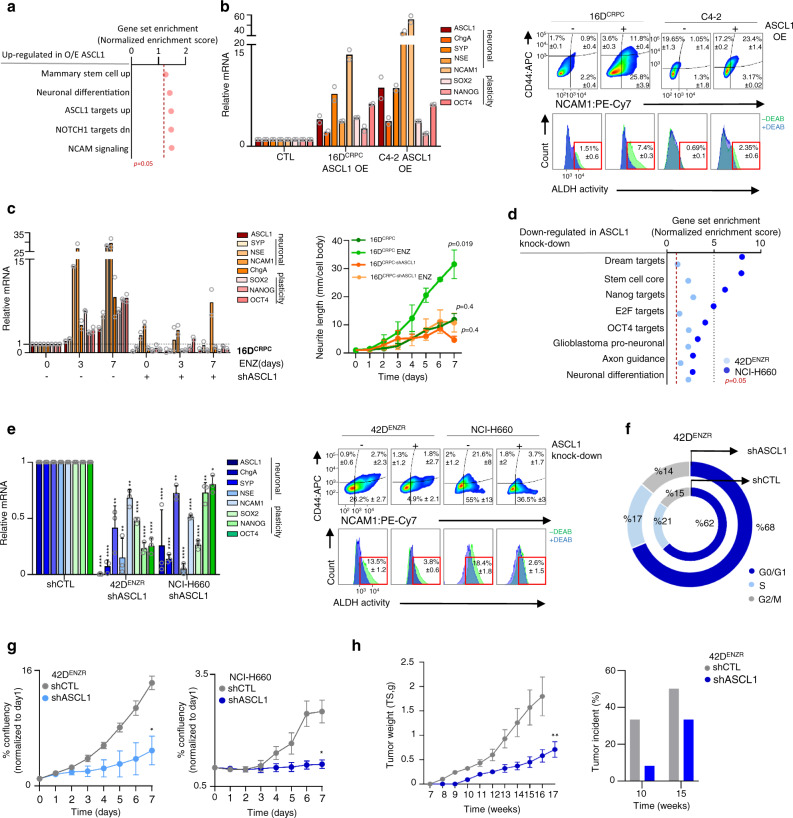
ASCL1 is a potent regulator of neuronal stem cell-like phenotype in prostate cancer. **a** GSEA in 16D^CRPC^ cells over-expressing (OE) ASCL1. Dotted line represents *p* = 0.05, statistical analysis was performed using a hypergeometric test (16D^CRPC^ CTL *n* = 3 and ASCL1 OE *n* = 1 biologically independent samples). **b** Neuronal and plasticity genes mRNA expression reported as mean of replicates normalized to GAPDH (*n* = 2 biologically independent samples) (left) NCAM1 and CD44 expression reported as mean of replicates (*n* = 2 biologically independent samples) (right top) ALDH activity reported as mean of replicates (n = 2 biologically independent samples) (right bottom) in CRPC over-expressing ASCL1. See also Supplementary Fig. 3. **c** 16D^CRPC^ shASCL1 treated with ENZ reported as mean of replicates (*n* = 2 biologically independent samples) (left) Neuronal-like morphology reported as mean ± SD with significance evaluated at endpoint, (*n* = 3 biologically independent samples; two-tailed unpaired t-test) (right). **d** GSEA in 42D^ENZR^ shASCL1 and NCI-H660 siASCL1. Dotted line represents *p* = 0.05, statistical analysis was performed using a hypergeometric test. **e** Neuronal and plasticity genes mRNA expression normalized to GAPDH (left) NCAM1 and CD44 expression (right top) ALDH activity (right bottom) in NEPC shASCL1 reported as mean ± SD (42D^ENZR^ *p-*value of ASCL1 < 0.000001, CHGA = 0.000009, SYP = 0.006, NSE = 0.007, NCAM1 = 0.001, SOX2 = 0.000003, NANOG = 0.00001, OCT4 = 0.00004 and NCI-H660 *p-*value of ASCL1 < 0.000001, CHGA = 0.000002, SYP = 0.001, NSE < 0.000001, NCAM1 = 0.000004, SOX2 = 0.000006, NANOG = 0.006 and OCT4 = 0.01; two-tailed unpaired *t*-test; *n* = 3 biologically independent samples). See also Supplementary Fig. 3. **f** Cell cycle phases comparing 42D^ENZR^ shASCL1 vs shCTL reported as mean in percentage (*p-*value of G0/G1 = 0.04, S = 0.006 and G2/M = 0.3; two-tailed unpaired t-test, n = 3 biologically independent samples). **g** Proliferation of 42D^ENZR^ (left) NCI-H660 (right) following shASCL1 reported as mean ± SD, with significance evaluated at the end point (*p-*value of 42D^ENZR^ =  0.003 and NCI-H660 = 0.019; two-tailed unpaired *t*-test, n =  3 biologically independent samples). See also Supplementary Fig. 3. **h** 42D^ENZR^ tumor size reported as gram (gr) (left) tumor intake shown as percentage of mice developed tumors post injection (right) in shASCL1 vs shCTL reported as mean ± SEM, with significance evaluated at the end point, *p* = 0.0056, shASCL1 *n* = 5 and shControl *n* = 6 biologically independent animal; two-tailed unpaired *t*-test. See also Supplementary Fig. 3. Source data are provided as a Source Data file.

To further explore the biological function of this transcription factor, ASCL1 was overexpressed in 16D^CRPC^ and RNAseq was performed, first we validated the upregulation of ASCL1 expression and activity (Supplementary Fig. 3a) and found that ASCL1 induced stem and neuronal programs (Fig. 3a). Importantly, ASCL1 was sufficient to increase NE and CSC markers (Fig. 3b, left panel), which were enhanced with ENZ treatment (Supplementary Fig. 3c), similar results were observed in lung adenocarcinoma (Supplementary Fig. 3d). ASCL1 induces NCAM1 and CD44 hybrid cell population and aldehyde dehydrogenase (ALDH) activity (Fig. 3b, right panel). These data suggest that ASCL1 alone is sufficient to induce cell plasticity and neuroendocrine phenotype. To determine whether ASCL1 is required for the development of neuronal and stem cell-like phenotype, knockdown of ASCL1 in 16D^CRPC^ prevented the ENZ-induced upregulation of CSC and NE markers (Fig. 3c) as well as neuronal-like morphology (Supplementary Fig. 3e), and prevented ENZ-mediated upregulation of NCAM1 and CD44 (Supplementary Fig. 3f). Similar results were observed in C4-2 cell line (Supplementary Fig. 3g). These data suggest that ASCL1 is a requirement for neuronal and stem cell differentiation and functions to bias the cell fate towards neuronal and stem cell-like lineage, similar to what has been previously reported in pericyte to neurons re-programing^24^.

To evaluate the importance of ASCL1 in maintaining the neuronal phenotype and the plastic state, ASCL1 was silenced in 42D^ENZR^ and NCI-H660 cell lines. Gene set enrichment analysis revealed that loss of ASCL1 expression suppress pathways related to proliferation, stemness and neuronal development (Fig. 3d). Specifically, ASCL1 knockdown downregulates both CSC and NE genes expression following ASCL1 silencing using shRNA (Fig. 3e), siRNA or CRISPR in 42D^ENZR^ and NCI-H660 (Supplementary Fig. 3h), surface markers NCAM1 and CD44, ALDH activity (Figs. 3e) and 3D spheroids as a measure for functional properties of stemness (Supplementary Fig. 3i). These observations were further validated by western blot (Supplementary Fig. 3j). Moreover, ASCL1 knockdown in 42D^ENZR^ resulted in ~6% increase in G0/G1 population (Fig. 3f), and a decrease in cell proliferation capacity in vitro in NEPC cell lines 42D^ENZR^ and NCI-H660 using shRNA (Fig. 3g), CRISPR (Supplementary Fig. 3k), or siRNA (Supplementary Fig. 3l). These data are in-agreement with GSEA in Fig. 3d showing a decrease in “dream targets” and “E2F targets” pathways. This reduction in proliferation rate was not due to an increase in apoptosis (Supplementary Fig. 3m). The reduced proliferation was translated in vivo, where 42D^ENZR^ xenografts bearing knockdown of ASCL1 grew at slower rate compared to the control group. In addition, we found that ASCL1 was required for tumor initiation measured by tumor intake ratio (Fig. 3h). Expression of NE genes was significantly lower in tumors with ASCL1 knockdown compare to control (Supplementary Fig. 3n).

### ASCL1 cistrome is enriched for stem cell and neuronal targets

**Fig. 4:**
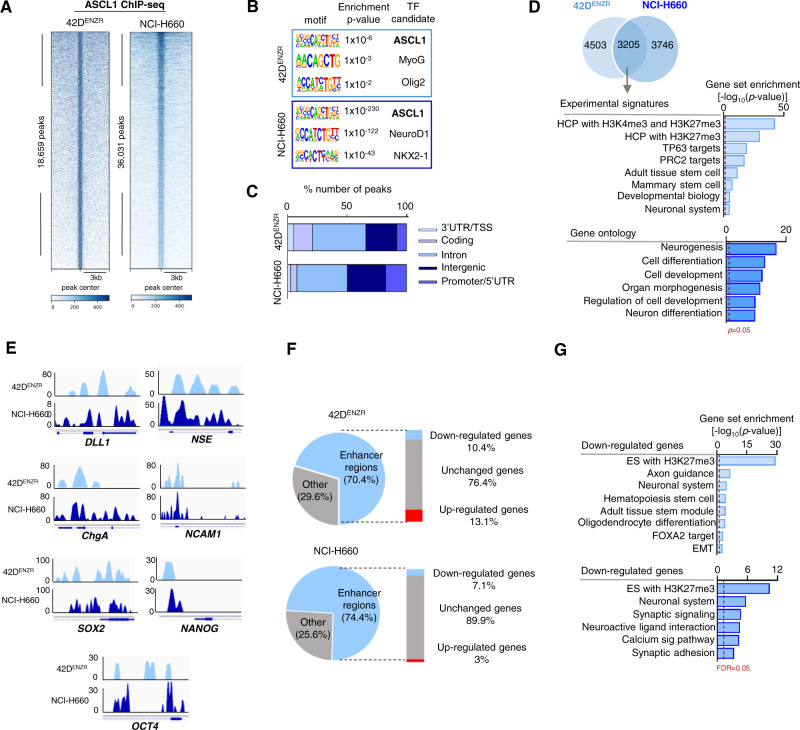
ASCL1 cistrome is enriched for PRC2 targets. **a** Heatmap of ASCL1 binding intensity in NEPC presented as fold change over input, with each horizontal line represent a 3 kb locus. 18,659 and 36,031 peaks were called in 42D^ENZR^ and NCI-H660, respectively. **b** Motif enrichment from ASCL1 ChIPseq in NEPC. Statistical analysis was performed using a cumulative hypergeometric test. **c** Genomic annotation of ASCL1 binding location in NEPC presented as percentage of total peaks. **d** Venn diagram showing overlapped genes in 42D^ENZR^ and NCI-H660 (top) GSEA of ASCL1 bound genes common between 42D^ENZR^ and NCI-H660. Dotted line represents *p* = 0.05, statistical analysis was performed using a hypergeometric test. **e** Visualization of genomic loci of NE and CSC markers using IGV showing relative occupancy of ASCL1 over input. **f** Pie chart showing the percentage of ASCL1 peaks at enhancer regions (defined as intergenic and intronic) in 42D^ENZR^ and NCI-H660 cell lines (left) bar chart showing the percentage of up- or down-regulated genes annotated to enhancers in ASCL1 knockdown (right). **g** Pathways associated to down-regulated enhancers in 42D^ENZR^ shASCL1 and NCI-H660 siASCL1. Dotted line represents FDR = 0.05, *p* < 0.05, statistical analysis was performed using a hypergeometric test. See also Supplementary Fig. 4. Source data are provided as a Source Data file.

To begin decipher the role of ASCL1 in lineage programming, we mapped genome-wide occupancy of ASCL1, using chromatin immunoprecipitation sequencing (ChIPseq) in NEPC cell lines. We identified 18,659 and 36,031 regions bound by ASCL1 in 42D^ENZR^ and NCI-H660 cell lines, respectively (Fig. 4a). As expected, ASCL1 binding was predominately centered on its canonical E-box binding motif (Fig. 4b). ASCL1 bound regions corresponded largely to enhancer (intronic and intergenic) regions (Fig. 4c), consistent with previous reports in glioblastoma and normal neurons^25,26^.We identified 3,205 ASCL1 bound genes common between the two cell lines and as expected, pathway analysis identified ASCL1 bound genes to be involved in stem and neuronal programming in NEPC cell lines (Fig. 4d). Visualization of genomic loci from ChIPseq demonstrated direct regulation of both CSC genes including *SOX2* (reported to be regulated by ASCL1, in small cell lung cancer (SCLC)^27^), *NANOG* and *OCT4* (encode by POU5F1); and NE genes including *CHGA*, *ENO2* (*NSE)*, *NCAM1*, *DLL1* (known ASCL1 target^28^) (Fig. 4e). Further supporting the direct regulation of CSC and NE genes by ASCL1, the expression of these genes was downregulated upon ASCL1 knockdown (Fig. 3e, Supplementary Figs. 3h and 4a). Accordingly, we sought to investigate the distinct ASCL1 cistrome and its association with NEPC programing. We integrated ASCL1 cistrome data with matched RNAseq. We identified enhancer regions bound by ASCL1 that lost their corresponding gene expression following ASCL1 knockdown, subsequent pathway analysis revealed loss of stem and neuronal programming following knockdown of ASCL1 within these NEPC enhancer regions (Fig. 4f). Notably, these enhancers were upregulated after overexpression of ASCL1 in 16D^CRPC^ (Supplementary Fig. 4b). These findings suggest that ASCL1 cistrome may function to unlock the lineage plasticity and further confirming the association between ASCL1 and the development of stem cell and neuronal phenotype.

### ASCL1 is required for EZH2 cistrome reprogramming

**Fig. 5:**
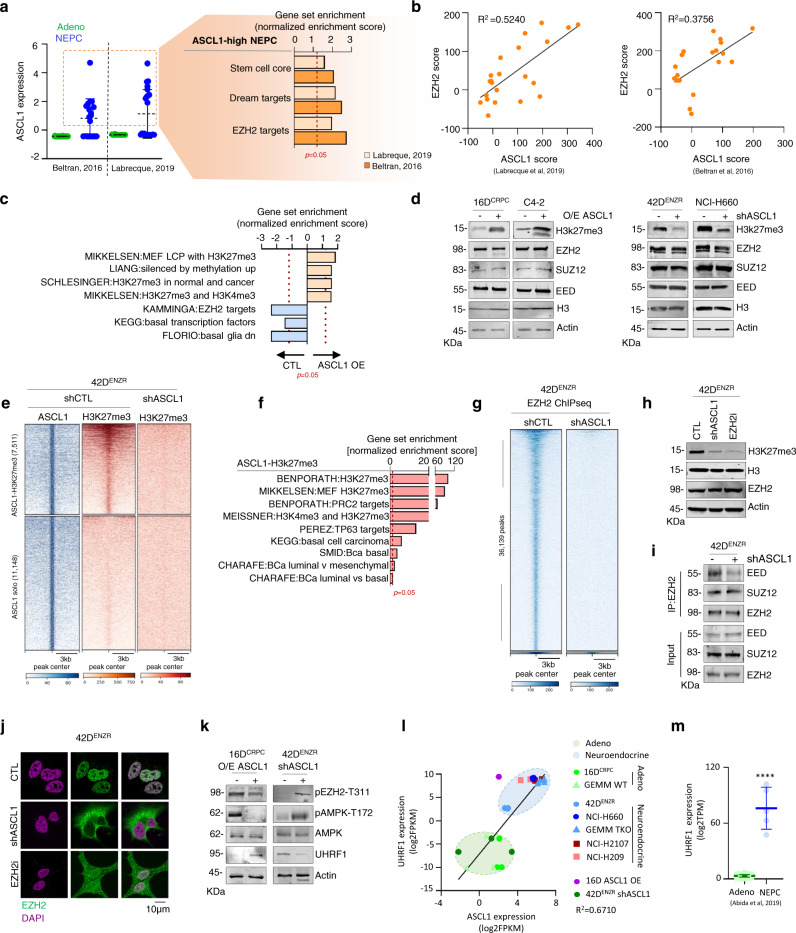
ASCL1 knockdown pheno-copies EZH2 inhibition. **a** ASCL1 expression reported as log2TPM mean ± SD in Beltran and Labrecque datasets^12, 15^ (left) GSEA in ASCL1-high vs ASCL1-low NEPC. Dotted line represents *p* = 0.05, statistical analysis was performed using a hypergeometric test (right). **b** EZH2 and ASCL1 score correlation reported as z-score, with each dot represents a patient tumor, *p* < 0.05, two-tailed unpaired t-test. See also Supplementary Fig. 5. **c** Pathways gain/loss in 16D^CRPC^ over-expressing ASCL1. Dotted line represents *p* = 0.05, statistical analysis was performed using a hypergeometric test. **d** Western blot shows expression of polycomb repressive complex 2 (PRC2) members and its activity as measured by H3K27me3 expression in castration-resistance prostate cancer (CRPC) over-expressing ASCL1 (left) and NEPC following knockdown of ASCL1 (shASCL1) (right), with actin and H3 expression used as loading controls (*n* = 3 biologically independent samples). **e** ChIPseq showing ASCL1 and H3K27me3 binding intensity reported as fold change over input. **f** Pathways associated with ASCL1-H3K27me3 co-bound regions. Dotted line represents *p* = 0.05, statistical analysis was performed using a hypergeometric test. See also Supplementary Fig. 5. **g** ChIPseq showing EZH2 binding intensity reported as fold change over input. **h** Western blot shows H3K27me3 in 42D^ENZR^ shASCL1 and EZH2 inhibition (EZH2i), with actin and H3 expression used as loading controls (n = 3 biologically independent samples). **i** PRC2 complex measured by co-immunoprecipitation in 42D^ENZR^ shCTL and shASCL1 (*n* = 3 biologically independent samples). **j** Visualization of nuclear localization of EZH2 in control vs cytoplasmic localization in shASCL1 or EZH2 inhibition (EZH2i), “EZH2” (green), “DAPI” (purple), scale bar=10 μm (*n* = 3 biologically independent samples). **k** UHRF1 expression and pAMPK-T172 in CRPC over-expressing ASCL1 (left) NEPC shASCL1 (right), actin was used as loading control (*n* = 3 biologically independent samples). **l** Correlation between expression of UHRF1 and ASCL1 in prostate and small cell lung cancer cell lines. Data reported as log2FPKM, with each dot represent one cell line. **m** Expression of UHRF1 in prostate cancer patients reported as log2FPKM mean ± SD, Adeno *n* = 155 and NEPC n = 5 patients, *p* < 0.0001; two-tailed unpaired t-test. Source data are provided as a Source Data file.

We observed that ASCL1 expression is heterogeneous in NEPC with two clusters (ASCL1-high and ASCL1-low) (Fig. 2g). Pathway analysis comparing ASCL1-high vs. ASCL1-low revealed that NEPC patients with high ASCL1 expression are enriched with pathways regulating plasticity and EZH2 activity (Fig. 5a). These data were supported by a strong positive correlation between ASCL1 and EZH2 expression (Supplementary Fig. 5a) and activity (Fig. 5b and Supplementary Fig. 5b, c) (R^2^ = 0.3756 in Beltran dataset^15^ and R^2^ = 0.5240 in Labrecque dataset^12^, *p*-value < 0.05). ENZ induced ASCL1 expression in CRPC concomitantly with increase EZH2 activity (Supplementary Fig. 5c, d). Interestingly, ASCL1 cistrome was enriched with PRC2 targets (Fig. 4d) and loss of ASCL1 decreased EZH2 activity in NEPC cell lines (Fig. 4f, g). Conversely, over-expression of ASCL1 in CRPC led to an increase in EZH2 activity, as measured by enrichment of pathways associated with histone 3 lysine 27 tri-methylation (H3K27me3), a surrogate measure of EZH2 activity (Fig. 5c and Supplementary Fig. 5e). These data were further validated at the protein level showing that ASCL1 overexpression increased H3K27me3, and conversely, knockdown of ASCL1 abrogates this histone mark (Fig. 5c; Supplementary Fig. 5f). This alteration of EZH2 activity by ASCL1 was not unique to prostate cancer, but seems like a common effect of ASCL1 as observed in lung adenocarcinoma (Supplementary Fig. 5c) and SCLC (Supplementary Fig. 5h). Expression of EZH2 or the PRC2 subunits SUZ12 and EED were not altered by ASCL1 overexpression or knockdown (Fig. 5c), suggesting that ASCL1 alters EZH2 activity. Overlaying ASCL1 and H3K27me3 ChIPseq revealed a 40% overlap which were lost after ASCL1 knockdown (Fig. 5d), were the number of H3K27me3 peaks went from 43,622 in control to 10,336 in knockdown ASCL1 (Supplementary Fig. 5i). Regions co-bound by ASCL1 and H3K27me3 were enriched with ASCL1 motif (Supplementary Fig. 5j). Of note, within same region DNA binding motif of TF OCT was enriched. This region was enriched for PRC2 targets as well as basal/luminal phenotype (Fig. 5g). Visualization of genomic loci of *TMPRSS2* and *ALDH1A3*, genes correlating with luminal phenotype in prostate cancer^29,30^, showed co-occupation of ASCL1 and H3k27me3 on promoter and gene body (Supplementary Fig. 5k).

To evaluate how loss of ASCL1 regulates the EZH2 cistrome, we performed ChIPseq for EZH2 in 42D^ENZR^ cell line following knockdown of ASCL1. Unexpectedly, comparative analysis between control and ASCL1 silencing condition revealed a significant loss of EZH2 binding to chromatin (36,139 peaks in control vs 293 in ASCL1 knockdown) (Fig. 5h). We found that ASCL1 knockdown decreased H3K27me3 to comparable level as targeting EZH2 (EZH2i) enzymatic activity using GSK126 (Fig. 5i). This loss of H3K27me3 was due to the disruption of PRC2, where EED was pulled down at considerably lower rate after ASCL1 silencing compared to control (Fig. 5j) similar to inhibiting EZH2 with GSK126^31^. ASCL1 knockdown induces EZH2 accumulation in the cytoplasm (Fig. 5k) and its phosphorylation on threonine 311 (pEZH2-T311) (Fig. 5k), known to play an important role in EZH2 function and localization^32^. EZH2 phosphorylation on T311 has been reported to be regulated by AMPK, we found that ASCL1 knockdown increased AMPK activity as measured by its phosphorylation on T172 (Fig. 5k). Interestingly, AMPK activity has been recently shown to be controlled by the nuclear factor UHRF1 (a key epigenetic regulator that bridges DNA methylation and chromatin modification^33^) in diverse cell models^34^. We found that ASCL1 positively regulates UHRF1 expression (Fig. 5k and Supplementary Fig. 5l) by binding upstream of its promoter in prostate^35^ and small cell lung cancer^20^ (Supplementary Fig. 5n). Expression of UHRF1 and ASCL1 significantly correlates in NEPC patients^12^, in prostate and small cell lung cancer cell lines as well as GEM model^14^ (Fig. 5l and Supplementary Fig. 5m). UHRF1 expression is significantly higher in NEPC tumors compared to adenocarcinoma^16^ (Fig. 5m). Together we identified a pathway by which ASCL1 regulates UHRF1, the gate keeper of AMPK, to regulate EZH2 methyl-transferase activity.

### Loss of ASCL1 initiates a lineage switch from neuronal to luminal

**Fig. 6:**
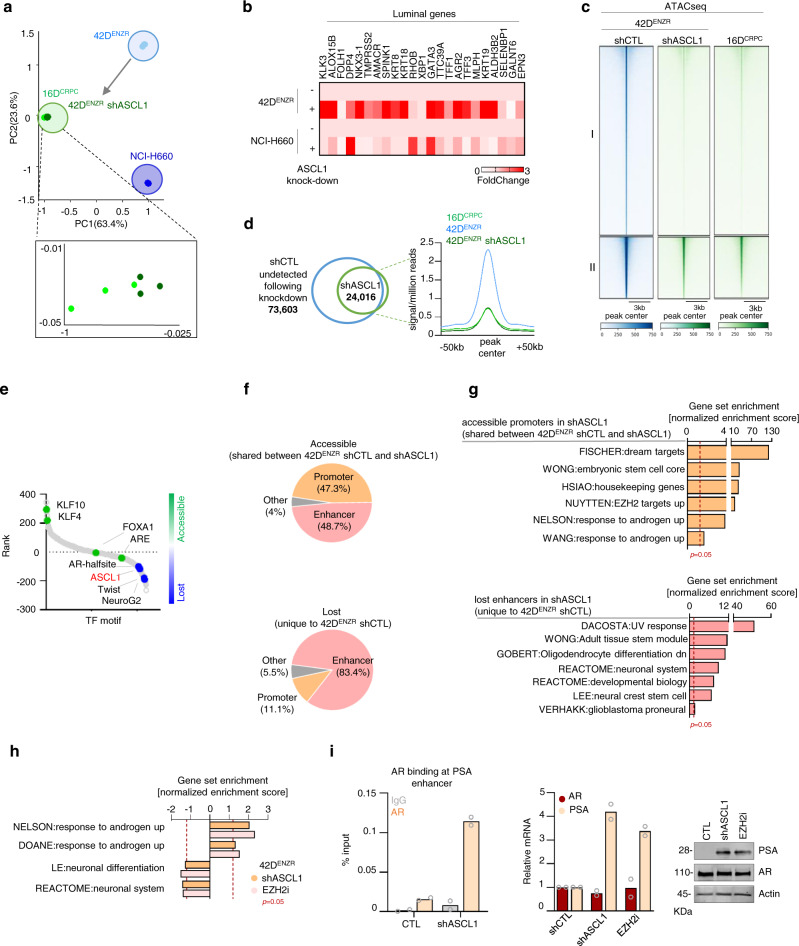
Loss of ASCL1 re-activates the luminal lineage. **a** Global transcriptome profiling of the indicated cell lines presented as principal component analysis (PCA) (*n* = 3 biologically independent samples). **b** Expression of luminal markers in NEPC following ASCL1 knockdown reported as fold change over control (Data reported as mean of replicates. 42D^ENZR^ shCTL and shASCL1 *n* = 3 and NCI-H660 CTL and siASCL1 *n* = 1). **c** Heatmap of accessible region in 42D^ENZR^ control, shASCL1 and 16D^CRPC^ represented as 3 kb window around the peak center. See also Supplementary Fig. 6. **d** 73,603 accessible regions identified in 42D^ENZR^ control vs 24,016 in shASCL1 (left) reported as average accessibility signal profile (right). **e** Ranked transcription factor motif comparing regions remained accessible in shASCL1 vs. regions unique to shControl, ranked based on differential *p*-value. Statistical analysis was performed using a hypergeometric test. **f** Genomic annotation shown as percentage of all peaks. **g** Pathways associated with shared accessible promoters (top) unique lost enhancers (bottom). Dotted line represent *p* = 0.05, statistical analysis was performed using a hypergeometric test. **h** GSEA of pathways up- or down-regulated in 42D^ENZR^ shASCL1 or EZH2 inhibition (EZH2i). Dotted line represents *p* = 0.05, statistical analysis was performed using a hypergeometric test. See also Supplementary Fig. 6. **i** Androgen receptor (AR) ChIP-PCR shows AR binding to prostate specific antigen (PSA) enhancer region (*n* = 2 biologically independent samples) (right) mRNA expression of PSA normalized to GAPDH (*n* = 2 biologically independent samples) (middle) and western blot shows protein expression of PSA, with actin as loading control (right) (*n* = 3 biologically independent samples). Source data are provided as a Source Data file.

ASCL1 knockdown in 42D^ENZR^ led to a change in the transcriptome similar to those observed in 16D^CRPC^ (Fig. 6a) with increase in the expression of luminal genes in 42D^ENZR^ and NCI-H660 following knockdown of ASCL1 (Fig. 6b). Of note, these luminal genes are co-bound by ASCL1 and EZH2 and are methylated at promoter region in 42D^ENZR^ cell line (Supplementary Fig. 6a). These data support the notion that ASCL1 knockdown re-activates the luminal lineage and the conversion of lineage to an AR-dependent state. These data are supported with analysis of the chromatin landscape using ATACseq, where widespread chromatin remodeling was observed after loss of ASCL1 in 42D^ENZR^ (73,603 peaks in control vs 24,016 peaks in ASCL1 knockdown) (Fig. 6c, d). In agreement, quantification of chromatin condensation showed an increase in chromatin compacting after knockdown of ASCL1 in 42D^ENZR^ (Supplementary Fig. 6b). Comparative analysis of accessibility between 42D^ENZR^ control and ASCL1 knockdown showed that ASCL1 knockdown change the resembling to CRPC. In support, accessible regions shared between 42D^ENZR^ control, ASCL1 knockdown and 16D^CRPC^ (Fig. 6c, region II) were significantly less accessible in 42D^ENZR^ ASCL1 knockdown and CRPC (Fig. 6c, d). Regions unique to 42D^ENZR^ (Fig. 6c, region I) were slightly more accessible in ENZ-treated CRPC (Supplementary Fig. 6c). Motif enrichment analysis identified loss of accessibility at number of DNA-binding motif of neuronal TF including ASCL1, while TF KLF was among the highly accessible TF following ASCL1 silencing (Fig. 6g). Interestingly, regions that lost accessibility (Fig. 6c, region I) were associated with enhancer regions regulating stem and neuronal programing, while conversely, regions that remained accessible (shared between 42D^ENZR^ control and ASCL1 knockdown) (Fig. 6c, region II) were equally mapped to promoter and enhancer regions enriched for various biological function such as housekeeping, proliferation and canonical AR signaling (Fig. 6f and g). These data support that ASCL1 is crucial in maintaining the chromatin architecture that is required for stem/neuronal phenotype. Of significance, H3K27me3 ChIPseq from the matching cell lines, identified shared regions carrying H3K27me3 histone mark only in 42D^ENZR^ and absent in 16D^CRPC^ and 42D^ENZR^ ASCL1 knockdown (Supplementary Fig. 6d), corresponding to increase protein level of H3K27me3 in 42D^ENZR^ compare to 16D^CRPC^ (Supplementary Fig. 6e). Interestingly, we observed a different pattern of histone methylation between these regions (Supplementary Fig. 6f). Knockdown of ASCL1 pheno-copies EZH2 inhibition leading to a decrease of NE pathways and re-activation of pathways implicated in canonical AR signaling (Fig. 6h). This lineage reversal was measured by increased of AR binding at *KLK3* (coding prostate-specific antigen (PSA)) enhancer resulting in re-expression of PSA (Fig. 6i and Supplementary Fig. 6g) with concomitant loss of expression in NE markers (Supplementary Fig. 6h), similar to EZH2 inhibition^31^. Together, we have shown that the large-scale chromatin remodeling induced in t-NEPC was lost following knockdown of ASCL1 leading to a switch in the lineage toward a luminal one and support the notion that the neuronal phenotype induced by ENZ can be reversed by targeting ASCL1, at least in a lineage plastic state.

## Discussion

The implementation of next-generation androgen receptor pathway inhibitors such as abiraterone and enzalutamide have increased the survival of patients with metastatic castrated resistant prostate cancer^1,2^. These agents maintain the ability to blunt AR signaling. However, prolonged AR pathway inhibition can alter the archetypal course of the disease, leading to histological dedifferentiation and alterations in cell lineages including the aggressive treatment induced neuroendocrine phenotype^3,15,36^. Importantly, these therapies are now used earlier in clinical management for patients with aggressive localized prostate cancer^37,38^. While follow-up on these trials are still limited, it is reasonable to speculate that the use of potent ARPIs may lead to higher incident of treatment induced NEPC. Therefore, the need for new therapies centered on targeting lineage plasticity and neuroendocrine differentiation is paramount.

In this study, we dissected the early epigenetic and transcriptional events regulating the trans-differentiation of CRPC to NEPC in response to ARPIs. Analysis of the CRPC transcriptome and chromatin architecture following ENZ treatment revealed an acute luminal-to-neuroendocrine lineage switch. Particularly, suppression of canonical AR signaling was concomitant with synchronized activation of stemness and neuronal lineage programs. We identified ASCL1 as one of the top enriched motifs in CRPC following ARPIs, which was also observed in NEPC cells, in GEM models with *MycN* overexpression in the context *of PTEN and RB1* deletion^39^ and in luminal prostate cells transformed to neuroendocrine with *Myc*, *Akt* and *Bcl2* in the context of *RB1* and *TP53* deletion^40^. These data suggest that determination of alternative cell fates is decided at the chromatin level early during the evolution of CRPC to NEPC and emphasizes the power of ARPIs in driving this process.

We report here that remodeling of the chromatin by ARPIs was coupled with increase expression and activity of ASCL1. High levels of ASCL1 were observed in subset of NEPC patients (Fig. 2f, g; and Supplementary Fig. 2i, j)^41^, SCLC^42^ and GBM^43^. ASCL1-low NEPC can be driven by other neuronal transcription factors such as NeuroD1, YAP1 or POU2F3 similar to what was observed in SCLC^39,44–46^ Building on the observation that ASCL1 induces rapid neurogenesis during normal neuron development^47,48^, neuronal differential in glioblastoma stem cell^25^ and neuroendocrine differentiation in lung cancer^49^ and induces NE markers in prostate cancer^50,51^, we found that ASCL1 induces neuroendocrine phenotype by directly regulating neuronal and stem cell programs. Significantly, we identified that ASCL1 binds to PRC2 targets and regulates EZH2 activity. Mechanistically, ASCL1 through direct transcriptional regulation of UHRF1, the AMPK gatekeeper, mediates AMPK inactivation independently of AMPK upstream kinases models^34^. UHRF1 directly binding to AMPK and recruits the phosphatase PP2A complex to trigger AMPK T172 de-phosphorylation^34^; hence, stabilizes the PRC2 complex and increases H3K27 tri-methylation. Conversely, ASCL1 knockdown inhibits EZH2 activity. These resulted to a shift in the chromatin landscape back to a CRPC-like state and allowed the conversion of the neuroendocrine to luminal lineage. These data suggest that ASCL1 and EZH2 may represent a molecular conduit that contributes to lineage plasticity and treatment resistance. In support of this concept, high levels of EZH2 were reported in NEPC^14,52^ and its expression was required for the acquisition of lineage plasticity and neuroendocrine differentiation post ARPIs^31^.

ASCL1 plays a central role in promoting and maintaining neuronal stem cell fate. We found that loss of ASCL1 switches the cell lineage to a luminal state by modulating genome-wide chromatin remodeling. In recent years, a number of clinical studies have focused on targeting epigenetic factors in prostate cancer, including EZH2, in combination with hormone therapy^53^. Our work provides basis for targeting transcription factor ASCL1 in the lineage plastic neuroendocrine-like tumors, to induce similar downstream effect as envisage for targeting EZH2 and provides a proof of principle that in highly plastic AR positive t-NEPC seen in the clinic^3^ patients may benefit from targeting ASCL1 or EZH2 to reverse the neuroendocrine phenotype to an alternative lineage and re-addict tumors to ARPIs.

In closing, we report a role for pro-neuronal transcription factor ASCL1 in modulating the chromatin dynamics to support a plastic lineage by orchestrating early chromatin events and regulatory networks that determine a neuronal stem cell-like lineage commitment. In the treatment-resistant, high plasticity state inhibition of ASCL1 reverses the lineage switch to epithelial-luminal, providing a potential for targeting these highly aggressive tumors. Similar to NEPC, a subset of glioblastoma and small cell lung cancers are defined by elevated expression of ASCL1. This work provides much-needed insight into ASCL1 function and dependency that together nominates ASCL1 as a bona fide clinical target.

## Methods

### Cell lines and tissue culture

NCI-H660 (cat. #CRL-5813), C4-2 (cat. #CRL-3314), A549 (cat. #CRM-CCL-185) and H2107 (cat. #CRL-5983_FL) cell lines were obtained from ATCC. HEK293T (cat. #R70007) were obtained from ThermoFisher. CRPC (16D^CRPC^) and ENZ-resistant AR+ NE-like (42D^ENZR^) cell lines were generated from LNCaP cells, previously detailed by our group^31,54^. We utilized an in vivo model of CRPC and ENZ resistance previously developed by us^31,54,55^ that mirrors clinically reported treatment refractory phenotypes. LNCaP cells were inoculated into mice and upon castration CRPC tumors (16D^CRPC^) emerged. Further treatment of 16D^CRPC^ tumors with ENZ (10 mg/kd/d) lead to the re-emergence of tumors with heterogeneous resistance mechanisms, including lineage plasticity. 42D^ENZR^ tumors exhibit AR expression, but loss of canonical AR signaling, concomitant with an enrichment in plasticity and neuronal transcriptional programs. NCI-H660 and NCI-H2107 cell lines were cultured in HITES medium following ATCC recommendation. LNCaP-driven cell lines and A549 were cultured in RPMI-1640 media plus 5% FBS (FBS; Gibco, cat. #A3160701). ENZ-resistant cell lines were cultured in 10µmol/L ENZ. HEK293T cells were cultured in DMEM media supplemented with 10% FBS and 2mM L-glutamine. When indicated, cells were treated with 10µmol/L of ENZ or EZH2 inhibitor GSK126 (Millipore, cat. #500580). Cultures were assessed for mycoplasma monthly, and all cell lines have been authenticated by short tandem repeat profiling.

### In vivo study

All animal studies were performed in accordance with protocols approved by the Animal Care Committee at the University of British Columbia (A16-0246). Mice were maintained in ventilated cages (4 mice per cage), with constant humidity of 25–47% and temperature of 21–22 degrees Celsius under 12hrs:12hrs dark/light cycle with ad libitum access to rodent chow diet and drinking water. Immunocompromised male mice (Envigo; strain: NU-Foxn1nu; 6-8 weeks old) were subcutaneously injected with 2 × 10^6^ cells per one site-right flank. When tumor reached ~150 mm^3^ they were randomly assigned to treatment groups (6 mice in each group). Mice were given 0.5% methyl cellulose or ENZ (10 mg/kg) administered by oral gavage 3 times per week starting from the day of injection. Tumor volumes was monitored and were measured twice weekly in a blinded fashion, and calculated with the formula volume V = (π(length x width x height))/6. When tumor volume reached 10% of body weight or a body weight loss > 15%, tumors were harvested for downstream analysis Mice were euthanized using isofluerane inhalant anesthesia followed by carbon dioxide euthanasia as described in University of British Columbia SOP ACC 03-2012 “Euthanasia of adult rodent using inhalant anesthetic followed by carbone dioxide”.

### Plasmids

The plasmid used to overexpress ASCL1 in our study, pPB[Exp]-Puro-EF1A > hASCL1, was constructed and packaged by VectorBuilder with vector ID is VB180704-1067ybm; for knockdown of ASCL1 our study used pLV[shRNA]-Puro-U6 > hASCL1[shRNA#1], with vector ID VB180704-1064qqg, which can be used to retrieved detailed information about the vector on vectorbuilder.com. Lentiviral packaging plasmids pMD2 (Addgene, cat. #8454) and psPAX7 (Addgene, cat. #12260) are available on Addgene. Plasmids used for CRISPR/Cas9-mediated genomic editing were constructed using GeneArt CRISPR Nuclease Vectors (Thermo Fisher). Double stranded oligos encoding a target-specific crRNA were generated and cloned into the gRNA expression cassette: ASCL1 (F: 5′- GCGTTTGCAGCGCATCAGTT). gRNA expression cassette lentiCRISPR v2 plasmid was purchased from Addgene (cat. #52961). All plasmids were analyzed for correct insertion by Sanger sequencing before use.

### siRNA transfection and stable cell line generation

For generation of stable cell lines, cells were transfected with 5 µg plasmid using TransIT-2020 (Mirus) in Opti-MEM media (Gibco) for 24 hours. Cells were maintained under antibiotic selection with puromycin (for shASCL1 stable cell lines; 10 µg/mL).

For siRNA knockout experiments, cells were transfected with 20 nM ASCL1 siRNA and scrambled siRNA in Opti-MEM using Lipofectamine RNAiMAX (Thermo). Cells were incubated for 18 h with siRNA, followed by a 4 hour recovery in complete media prior to re-transfection for 4 h.

ASCL1 siRNA sequences (5′-GCGCGGCCAACAAGAAGAUGAGUAA-3′ and 5′-UUACUCAUCUUCUUGUUGGCCGCGC-3′, Thermo, cat. #HSS100744) and Scrambled sequences (5′-AUCAAACUGUUGUCAGCGCUG, Dharmacon).

For overexpression of ASCL1 5 µg plasmid was transfected using Mirus T20/20 and OPTI-MEM media (Gibco) according to manufacturer’s instructions. 24 hours later OPTI-MEM media was replaced with complete media.

### CRISPR/Cas9-based gene editing

For generating stable 42D^ENZR^ with knockout ASCL1, cells were infected with TLCV2 vector (Addgene, cat. #87360) containing an ASCL1-specific guide RNA (GCGTTTGCAGCGCATCAGTT). A non-targeting gRNA (GTATTACTGATATTGGTGGG) was used as a control. At 5 days post-transfection, cells expressing Cas9/gRNA plasmid were isolated by FACS, seeded at single-cell density, and expanded. PCR of genomic DNA from individual clones was performed with primers flanking the gRNA target site and assayed by Sanger sequencing.

### Lentiviral vector preparation and infection

HEK293T cells were plated at 80% confluency 1 day prior to transfection in complete medium. Cells were transfected with ASCL1 plasmid, pMD2.G, and psPAX7 at 4:1:2 ratio. Media was changed 5 hours following transfection to complete medium. Virus was collected at 48 hours post transfection using 0.45uM Sterifilip. For transduction, 3 million cells were plated in 6 well plate in 1 mL appropriate standard serum-free media for the cell type, supplemented with 8ug/ml polybrene. 800 uL of viral supernatant was added to each cell. 24 hours later, cells were transferred into 10 cm plate, and media was replaced with complete medium. 0.5 ug/mL puromycin was added to cells for selection.

### Western Blot

Total proteins were extracted from adherent cells grown in vitro. Cells were washed with PBS and lysed in RIPA buffer (Thermo, cat. #PI89901) supplemented with 1x concentration of cOmplete EDTA-free protease inhibitors cocktail (Roche, cat. #11836170001) and phosphatase inhibitors (PhosSTOP, Roche, cat. #4906845001). Once protein concentrations were measured by using BCA protein assay (Thermo, cat. #23225) samples were boiled for 5 minutes is SDS sample buffer and ran on 10% or 15% SDS-PAGE gel depend on the molecular size of the target protein. Immunoprecipitation was performed using ImmunoCruz™ IP/WB Optima B System (Santa Cruz cat. #sc-45039) based on the manufacturer’s guideline. Transfer was done onto PVDF membranes (Millipore, cat. #IPVH00010) with pore size of 0.45um, blocked with Odyssey Blocking Buffer (LI-COR, cat. #15590545; 1:2), probed with primary antibodies at dilution indicated below. Membranes were imaged using the LI-COR Odyssey Imaging System with Li-COR Image Studio (version 4.2) software. Following parameters were used to scan the membranes: channels 700 for mouse and 800 for rabbit secondary antibodies; resolution of 169um; intensities of channel 700: 3.0 and channel 800: 1.5–3.0.

The following antibodies were used for immunoblotting: AR (Cell Signaling #5153; clone D6F11; 1:1000), AMPK (Cell signaling #2532S; 1:500), p-AMPK-T172 (Cell signaling, cat. #2535S; clone 40H9, 1:1000), ASCL1 (Santa Cruz, cat. #sc-374104; Clone D7, 1:200), ChgA (Abcam, cat. #ab151610; 1:1000), EED (Millipore, cat. #17-10034; 1:1000), EZH2 (Active Motif, cat. #39933; 1:2000), p-EZH2-T311 (Cell signaling, cat. #27888S: 1:1000), H3 (Cell signaling, cat. #14269s; clone 1B1B2, 1:2000), H3K27Me3 (Millipore, cat. #07-449; 1:2000), NSE/ENO2 (Agilent, cat. #M0873; 1:1000), OCT4 (Cell signaling, cat. #7546 S; clone D705Z; 1:500), PSA (Cell Signaling, cat. #5365; clone D6B1; 1:5000), SOX2 (Invitrogen, cat. #MA1-014; 1:500), SUZ12 (Cell Signaling cat. #3737S; clone D39F6; 1:1000), UHRF1 (Cell signaling, cat. #12387S: 1:1000). β-actin (Sigma #A2228; clone AC-74; 1:25,000) was used as loading control. IRDye 800CW donkey anti-Rabbit (LI-COR, cat. #926-32213; 1:10,000) and IRDye 680CW donkey anti-mouse (LI-COR, cat. #926-68072; 10,000) were used as secondary antibodies.

### Co-immunoprecipitation and immunoblotting

For immunoprecipitation, cells were washed with PBS and lysed in IP Lysis Buffer (Thermo, cat. #87787) supplemented with 1x cOmplete EDTA-free protease inhibitors cocktail (Roche, 11836170001) and phosphatase inhibitor (PhosSTOP, Roche, cat. #4906845001). Protein concentration was measured using Pierce BCA protein assay kit (Thermo, cat. #23225). 700ug sample was incubated overnight at 4°C with 20 μL magnetic A/G beads (Millipore) plus EZH2 (5 μg, Active Motif, cat. #39933) antibody. As a control, A/G beads were incubated with lysate and 1 uM IgG. After 24 hours beads were washed three times with IP lysis buffer and samples were eluted in sample buffer. Western blot was performed as previously explained.

### Quantitative real-time PCR (qRT-PCR)

RNA was extracted from cells using TRIZOL reagent (Invitrogen, cat. #15596026) following the manufacturer protocol. Reverse transcription was performed using SuperScript IV Reverse Transcriptase with random hexamers (Invitrogen, cat. #18090050). cDNA (60 ng/µl) was combined with FastStart TaqMan Probe Master and used as a template for TaqMan-based real-time PCR, using the following probes: AR (Hs00171172_m1), ASCL1 (Hs00269932_m1), ENO2/NSE (Hs00157360_m1), GAPDH (Hs02786624_g1), KLK3/PSA (Hs00426859_g1), NCAM1(Hs00941830), CHGA (Hs00154441_m1), and SYP (Hs00300531_m1), SOX2 (Hs01053049_s1), NANOG (Hs02387400_g1), and POU5F1(OCT4) (Hs04260367_gH). SYBR Green master mix was used with the following primers: UHRF1 forward (5′- CGA CGG AGC GTA CTC CCT AG) and reverse (5′- TCA TTG ATG GGA GCA AAG CA). *GAPDH* was used for normalization. Fold changes in mRNA expression levels were calculated using the comparative Ct method. Real-time PCR was performed using an ABI ViiA7.

### Flow cytometry and FACS

Cells were dissociated using Cellstripper™ (Corning®) at room temperature with gentle shaking and filtered through a 40-μm nylon cell strainer. Single cell suspensions were pelleted at 300 x *g* and re-suspended in flow cytometry buffer (2 mM EDTA, 1% FBS, 0.1% NaN_3_ in 1x PBS) with CD56/NCAM1, PE-Cyanine7-conjugated (Thermo, cat. #25-0567-42; 1:40) and CD44, APC-conjugated (Invitrogen, cat. #17-0441-82; 1:40) for 45 min at 4 °C. Cells were washed 2x with flow cytometry buffer, incubated with 7-AAD (Thermo, cat. #A1310) for 10 min at 4 °C (to exclude dead cells), and acquired on a FACS on Canti II (BD Biosciences). Data was analyzed using FlowJo software (version 10.4.2).

ALDH activity was measured using ALDEFLUOR kit (STEMCELL technologies, cat. #01700) following manufactures protocol.

Propidium iodide (Sigma, cat. #P4864) was used to stain for cell cycle analysis. Cells were fixed with cold 70% ethanol followed by suspension in 500 µl PI-solution in PBS containing 50 µg/ml PI, 0.1 mg/ml RNase A and 0.05% Triton X-100 and incubated for 40 min at 37 °C. Cells were washed 2x with flow cytometry buffer and acquired on a FACS on Canti II (BD Biosciences). Data was analyzed using FlowJo software (version 10.4.2).

### Immunofluorescence (IF)

24 hours before staining 50,000 cells were seeded on a cover slip in a 6-well plate. Cells were fixation in 4% PFA for 20 minutes (for chromatin condensation analysis prior to staining cells were treated with 3 uM Hoechst solution (ThermoFisher, cat. #33342) for 30 min at 37 °C). Cells were washed 3x in PBS and permeabilized in PBS with 0.1% saponin and 3% BSA for 1 h. Cells were incubated with primary antibody (EZH2, active motif, cat. #39933, 1:20) at 1:20 concentration for at 4 °C overnight. Cells were then washed 3 times with PBS the following day, and incubated in the secondary antibody, donkey anti-Rabbit IgG (H + L) Alexa fluor 488 (Invitrogen, cat. #A21206, 1:10,000), for 1 h at room temperature. Cells were washed 3 times with PBS, followed by incubation with DAPI (Thermo, cat. #D1306, 1:500). Cells were mounted on slides using mounding reagent (FluorSave Reagent, Millipore, cat. #345789). Fluorescent images were taken using 60x oil immersion objective using FV3000RS confocal microscope equipment using Olympus software FV31S-SW version 2.3.2.169. For EZH2 staining images were taken with following parameters: DAPI at 405 nm and EZH2 at 488.

For Hochst staining 8 images were taken at 1024 by 1024 pixels from each control and treatment at 10 µm scale. For chromatin condensation quantification ImageJ (version 1.8.0) was used to convert images to 800 by 800 pixel. Using Sobel edge detection tool in ImageJ the areas of condense chromatin within the nuclei of each cell was determined. The intensity of nuclear area was calculated and the edge were detected and quantified. These steps were then repeated for each image, average signal intensity was graphed comparing control and treatment cells^56^.

### Spheroid Assay

Single cells suspensions of cells (1,000 cells/ml) were plated on ultra-low attachment plates and cultured in serum-free NeuroCalt NS-A Basal Medium (Human) (STEMCELL cat. #05750), supplemented with 2% B27 (Thermo, cat. #A3582801), 1% N2 (Thermo, cat. #17502048), 20 ng/ml basic fibroblast growth factor (bFGF) (STEMCELL cat. #78003.1) and 20 ng/ml epidermal growth factor (EGF) (STEMCELL, cat. #78006.1), and 2ug/ml Heparin (STEMCELL, cat. #07980) for 7-10 days (First generation) and 7 days (Second generation). For serial passage, tumor spheres were collected using 40-μm cell strainers and dissociated with Accutase (STEMCELL, cat. #07922) for 10 min at room temperature to obtain single-cell suspensions. Tumor spheres were visualized by IncuCyte S3.

### Proliferation and NeuroTrack

2000 cells/well were seeded in a 96-well cell culture plates, allowed to attach overnight, treated with drug (when indicated), and imaged using the IncuCyte S3. Cell confluence was assessed using the IncuCyte Basic Analyzer with a minimum size filter of 400 µm^2^. Neuronal-like morphology was measured using the IncuCyte NeuroTrack software module (version 2018B) with the following settings: segmentation mode: brightness; segmentation adjustment: 0.5; minimum cell width: 15 µm; neurite filtering: better; neurite sensitivity: 0.4; neurite width: 4 µm. A minimum of 4 technical replicates, and 3 biological replicates, were performed for each cell line/treatment.

### RNA sequencing and data analysis

Total RNA was isolated from cell lines using the RNeasy Mini Kit (Qiagen, cat. #74104). Library constructions were performed using the NEBnext Ultra ii Stranded RNA Library Prep Kit, and sequencing was performed on an Illumina NextSeq500 (42x42bp paired-end reads).

Data was de-multiplexed using bcl2fastq2 Conversion Software (version 2.20) and the resultant read sequences were aligned to the hg19 human reference genome using STAR aligner^57^. Assembly and differential expression was estimated using Cufflinks software (version 2.2.1)^58^ available through the Illumina BaseSpace Sequence Hub. For patient tumors, sequencing data was aligned to hg38 using TopHat, and number of reads per gene were measured with HTSeq count. Gene counts (FPKM or TPM) were normalized using DESeq^59^ and subsequently log-transformed. For visualization purposes, the data were Z-transformed per gene. PCA plots were generated using ClustVis. Significance of expression level differences between pre- and post-treatment samples from the DARANA clinical trial was determined using a paired *t*-test.

### Chromatin Immunoprecipitation using sequencing (ChIPseq)

Cell lines were grown in media supplemented with 5% FBS (Gibco, cat. #A3160701) and processed for ChIP using the Magna ChIP Kit (Millipore, cat. #17-10085) according to manufacturer’s instructions with the following antibodies: ASCL1 (5 µg, Abcam, cat. #556604), EZH2 (5 μg, Active Motif, cat. #39933), H3K27Me3 (5 µg; Millipore, cat. #07-449). 10 million cells were fixed with 1% formaldehyde for 10 minutes at room temperature. Cells were collected. Chromatin was sonicated to approximately 500 bp. For ChIPseq, sequencing libraries (100 ng DNA per sample) were constructed using the KAPA HyperPrep Kit with Illumina TruSeq indexes (Roche, cat. #KK8510). Libraries were assessed for quality using gel electrophoresis, and libraries passing quality control (e.g., no primer dimers) were quantified using the KAPA Library Quantification Kit for Illumina (Roche, cat. #KK4824). Libraries were sequenced using the Illumina NextSeq500 (75 bp single-end reads).

For ChIP-PCR following antibody was used: AR (5 µg, Millipore, cat. #06-680), ASCL1 (10ug, Anti-MASH1 BD pharma, cat. #556604) and Flag-tag (DYDDDK Cell signaling, cat. #14793 S). The following IgGs were used: Rabbit IgG (Millipore, cat. #12-379) and Mouse IgG (Santa cruz, cat. #sc-2025). The following primer were designed around AR binding sites from 16D^CRPC^ and 42D^ENZR^ AR ChIPseq: KLK3/PSA enhancer region forward (5′ GCCTGGATCTGAGAGAGATATCATC 3′) and reverse (5′ ACACCTTTTTTTTTCTGGATTGTTG 3′). The following primers were designed around ASCL1 binding site from 42D^ENZR^ and NCI-H660 ASCL1 ChIPseq: CHGA enhancer region forward (5′-CTGACGTCATTTCCGGGGTC) and reverse (5′- CGTCTGTCGGTCGATCCTC), NCAM1 enhancer region forward (5′-TGCGAAGGCGTAGGGTAGAA) and reverse (5′- GGCTCTTTCACACGCAGTCT) and NSE enhancer region (5′- CGCACCTCTCCGCATCTC) and reverse (5′- AGGCGCAGTTAGAGATAGGC). Fold enrichment of regions relative to the input was evaluated by qRT-PCR as described above.

### Assay for Transposase-Accessible Chromatin using sequencing (ATACseq)

ATACseq experiments were performed as described^60,61^. Briefly, cells were collected by incubating in trypsin for 5 minutes at room temperature and subsequent centrifugation at 500 x *g* for 5 min at 4 °C. Cells were washed twice and re-suspended in PBS. 5 × 10^4^ cell pellet was used for tagmentation by incubating in 50 µl of 1x THS-seq buffer (25 µl 2x THS-buffer, 5 µl 10x Digitonin, 2 µl Illumina-TDE1) for 20 minutes at 37°C with 100xg in ThermoMixer (Eppendorf). To stop the tagmentation reaction, equal volume of 2x Tagmentation Stop Buffer [10 mM Tris-HCl (pH 8.0), 20 mM EDTA (pH 8.0)] was added to reaction and incubated for 10 min on ice. For cell lysis, equal volume of 2x Lysis Buffer [100 mM Tris-HCl (pH8.0), 100 mM NaCl, 40 µg/ml Proteinase K, 0.4% SDS] was added to the tagmentation mix and incubated at 65 °C for 15 min. The tagmented DNA library was purified in 20 µl EB using Qiaquick PCR purification kit (Qiagen). Number of amplification cycles and Library quantitation was done as described^60^ 75 bp paired-end sequencing was performed on an Illumina HiSeq2500.

### ChIPseq and ATACseq data analysis

FastQC version 0.11.9^62^ was used for quality control analysis of fastq files and in case of ATACseq fastq, adapter sequence were removed using Cutadapt v1.18. Raw reads were aligned to the hg38 (human) reference genome using BWA-MEM software (version 0.7.17)^63^ with default parameters. Alignments with mapping quality less than 60 were filtered out (leaving only uniquely mapped reads). The sam files were converted to bam files using Samtools software (version 1.1.2)^64^. Peaks were called using MACS2 (version 2.2.7.1)^65^ with FDR q-value = 0.05 using the narrow peak caller for accessible DNA, ASCL1 and EZH2, while the broad peak calling with –nomodel option was used for H3K27Me3. Deeptools v2.30.0^66^ program suit was used for visualization of data. Bedtools v2.28.0^67^ program suite was used to generate shared and unique peaks between various ChIPseq and ATACseq samples.

ATAC peaks were called from reads with a template length between 40 and 120 bp. Peaks were annotated using HOMER^67^. To compute the intersections between peak files, we used bedtools intersect program and two peaks were considered overlapping if the length of the overlapping region is greater than 50% of the length of either peak. For visualization, bigWig files were generated using deepTools software. Heatmaps were generated using *computeMatrix* with reference-point mode and *plotHeatmap* programs of deepTools.

### Motif analysis

MACS generated bed file after peak calling was converted to FASTA file with +/− 50 bp window around the center of each peak and Bedtools program suite for both ChIPseq and ATACseq peaks. The motif enrichment analysis was performed using FASTA file as input and findMotifs.pl HOMER program suit v4.10^68^. To identify significantly enriched motifs under a given condition, motifs were ranked by log *p*-value, and the difference in rank was plotted on a waterfall plot.

ASCL1 motif file was first generated using HOMER program suite, v4.11 (findMotifsGenome.pl program) for each accessible DNA peak (bed) file of interest. Then accessible DNA peak file was annotated using HOMER program suite (annotatePeaks.pl) along with the information about presence of ASCL1 motif. The annotated peak file was then parsed to retrieve only those accessible DNA features which contained ASCL1 motif using a custom AWK script. These parsed peak files were used to generate unique and shared bed files between samples of interest using Bedtools program suite v2.28.0 (bedtools intersect program). The intersected peak file of interest was annotated and annotation statistics was generated using HOMER program suite (annotatePeaks.pl). Annotation statistics data was plot to generate ASCL1 motif distribution. Annotated gene information data was used to perform GSEA and generate expression heatmap.

### PCA and PLSR analysis

Principal Component Analysis (PCA) was performed using the *prcomp()* function in R. The log2 transformed TPM values were used as input. Supervised partial least squares discriminant analysis (PLS-DA) was carried out to identify similarity between samples from multiple independent cell lines RNAseq using a multivariate integrative method, MINT, as part of the mixOmics R package.

### Gene signature scores

Previously described ASCL1^20^, AR^69^ and EZH2^70^ score were computed by the sum of Z-score transformed expression level across each score’s gene list. Adult Stem Cell (ASC)^13^ was computed using GSEA (v4.0.2).

To generate ASCL1 signature in prostate cancer we performed ChIPseq analysis on ASCL1 positive neuroendocrine prostate cancer cell lines 42D^ENZR^ and NCI-H660 (from our study and from Baca et al.^35^) as well as in LNCaP cells overexpressing ASCL1^35^. Several thousand ASCL1 bound sites were identified following analysis of each ChIPseq. A total of 2,360 genes annotated to ASCL1 were found common among the four samples. As expected, we identified ASCL1 binding at bona fide ASCL1 regulated genes such as *INSM1*, *ID4*, *HES6*, *CHGA* and *DLL4*. Suggesting that ASCL1 supports the neuroendocrine phenotype in NEPC. Integrated ASCL1 ChIPseq data with transcriptomics (RNAseq) data. To identify a consensus ASCL1 transcriptome we utilized RNAseq from publicly available ASCL1 (+) vs ASCL1 (−) patient driven xenografts^12^. 160 ASCL1-bound genes identified in prostate cancer ChIPseq studies also exhibited significantly higher mRNA expression (log2FoldChange > 2) in the ASCL1 (+) compared to ASCL1 (−) LuCaP PDXs. These genes represent targets of ASCL1, where expression is likely directly regulated by ASCL1 binding.

Scores were computed by the sum z-score transformed expression levels across each score’s gene list for patient data sets and computed the sum of log2FPKM transformed expression levels across each score’s gene list for cell lines.

### Gene ontology and pathway analysis

Pathway analysis using gene set enrichment analysis (GSEA) software from the Broad Institute (Massachusetts Institute of Technology) was used to identify functions of differentially expressed genes within the Molecular Signatures Database (MSigDB, version 7.1)^71,72^. The tool was run in classic mode to identify significantly enriched biology pathways. Pathways enriched with a nominal *p* value < 0.05 and false discovery rate (FDR) < 0.25 were considered to be significant. Single sample GSEA (ssGSEA) was carried out using gProfiler, a web server for functional enrichment analysis and conversion of gene lists^73^.

### Statistics and reproducibility

All statistical analysis and visualization was performed using GraphPad Prism (version 8), unless otherwise specified. False discovery rate (FDR) and p-value for all GSEA was carried out by GSEA software (version 7.1) or gProfiler web server^73^ and for motif analysis by HOMER using hypergeometric test. Representative data shown in micrographs such as western blot has been repeated 3 times with independent biological samples unless otherwise indicated. Data acquired from the IncuCyte S3 for proliferation or NeuroTrack analysis has been repeated with 3 independent biological samples. Representative data shown for all in vitro experiments were repeated at least two times unless otherwise indicated. In bar graphs, box and whisker, and violin plots, unpaired, two-tailed, student’s *t*-tests were performed to analyze statistical significance between groups using GraphPad Prism (version 8). For longitudinal profiling experiments, a two-tailed student’s *t*-test was performed to determine the statistical difference at the final time point using GraphPad Prism (version 8). *P*-value<0.05 was considered significant. Significance is indicated as follows in the figures: *P < 0.05; **P < 0.01; ***P < 0.001; ****, *P* < 0.0001. All exact p-values are listed in the corresponding Source Data.

### Reporting summary

Further information on research design is available in the Nature Research Reporting Summary linked to this article.

## Supplementary information

Supplementary Information

Description of Additional Supplementary Files

Supplementary Data 1

Supplementary Data 2

Supplementary Data 3

Supplementary Data 4

Reporting Summary

## Data Availability

RNAseq, ChIPseq and ATACseq data generated in this study has been deposited in the GEO database under the accession GSE183200. The publicly available RNAseq data used in this study from the SU2C/PCF-West Coast Dream Team cohort was downloaded from Aggarwal et al.^3^, the Beltran 2016 cohort from Beltran et al.^15^, the Labrecque 2019 from Labrecque et al.^12^ and the CALGB 90203 cohort from Beltran et al.^21^. The gene expression publicly available data of SKO/DKO/TKO prostate cancer GEMM dataset used in this study is available in the GEO dataset under the accession code GSE90891^14^. The publicly available LuCaP PDX RNAseq data used in this study is available in the GEO dataset under the accession code GSE126078^12^. The LT331 publicly available RNAseq used in this study is available in the GEO dataset under the accession code GSE41193^19^. The publicly available RNAseq of LNCaP cells with androgen deprivation used in this study are available in the GEO dataset under the accession code GSE8702^74^. 42D^ENZR^ EZH2, 42D^ENZR^ H3K27me3 ChIPseq, and SKO/DKO/TKO GEMM ATACseq data used in this study are publicly available in the GEO dataset under the accession code GSE138460^31^. The publicly available LNCaP over-expressing ASCL1 ChIPseq data used in this study was borrowed from Baca et al.^35^. The publicly available transformed prostate and lung epithelial ATACseq data used in this study are available in the GEO dataset under the accession code GSE118204^40^. The publicly available LuCaP PDX ATACseq data used in this study are available in the GEO dataset under the accession code GSE156291^44^. All source data are provided as a Source Data file with this paper. The remaining data are available within the Article, Supplementary Information, or Source Data file. Source data are provided with this paper.

## Source data

Source Data

## Code availability

Custom AWK codes generated for the purpose of this study are available in this link https://github.com/ZoubeidiLab/Qualityfilter_SAM/commit/f7bfcbb9d2525212c7190317ddb58db471e0b56a.
