# Supplementary material for: ASCL1 activates neuronal stem cell-like lineage programming through remodeling of the chromatin landscape in prostate cancer
Source: Nat Commun. 2022 Apr 27;13:2282. doi: 10.1038/s41467-022-29963-5 (PMC9046280; doi:10.1038/s41467-022-29963-5)

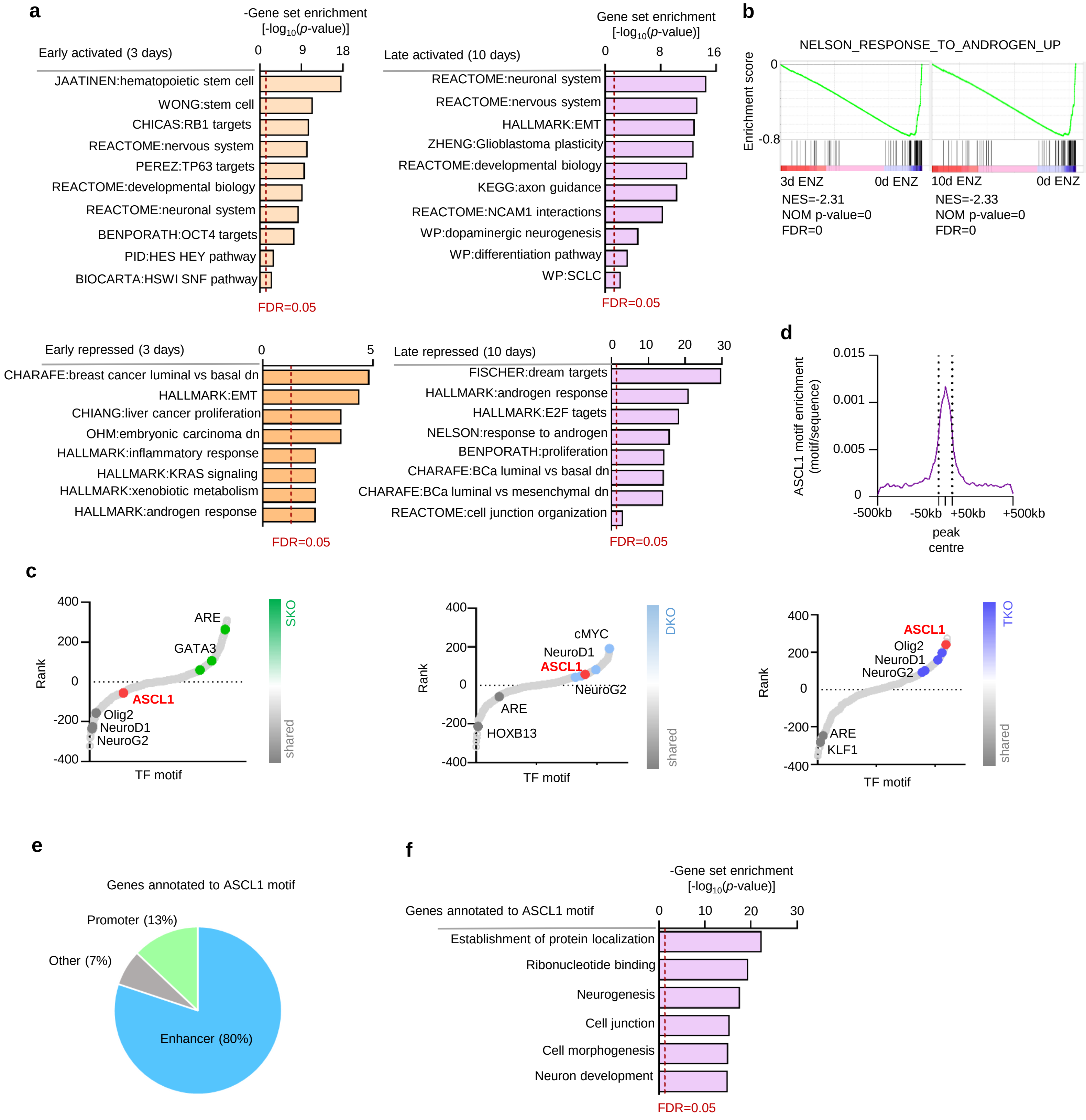

Supplemental Figure 1

a) Integrating ATACseq and RNAseq, gene set enrichment analysis (GSEA) was used to assess the transcriptional response to enzalutamide (ENZ) treatment in $16D^{CRPC}$ cells (3 or 10 days treated with ENZ vs. $16D^{CRPC}$ non-treated) presented as: early repressed; late repressed; early activated; late activated. Dotted line represent false discovery rate (FDR)=0.05 and $p<0.05$. Statistical analysis was performed using a hypergeometric test (n=1 biological sample).

b) GSEA shows loss of canonical androgen receptor (AR) signaling[76] after ENZ-treatment in $16D^{CRPC}$. $p<0.05$, statistical analysis was performed using a cumulative hypergeometric test.

c) Transcription factor (TF) binding motifs surrounding accessible chromatin in unique vs. shared regions, ranked based on differential p-value in PB-Cre4:Ptenf/f (single knockout (SKO)) and PB-Cre4:Ptenf/f;Rb1f/f (double knockout (DKO)) and Cre4:Ptenf/f;Rb1f/f;TP53f/f (triple knockout (TKO)) genetic engineered mouse models (GEMMs)[14]. Each dot represents a motif. Statistical analysis was performed using a cumulative hypergeometric test.

d) Enrichment of ASCL1 motif at accessible peak center (accessible region define as ± 50kb from peak center).

e) Genomic annotation for genes associated with ASCL1 motif shown as percentage of all peaks.

f) Gene set enrichment analysis shows Gene Ontology (GO) pathways associated to genes annotated to ASCL1 motif in 10 days ENZ treated $16D^{CRPC}$. Dotted line represents FDR=0.05 and $p<0.05$, statistical analysis was performed using a hypergeometric test.

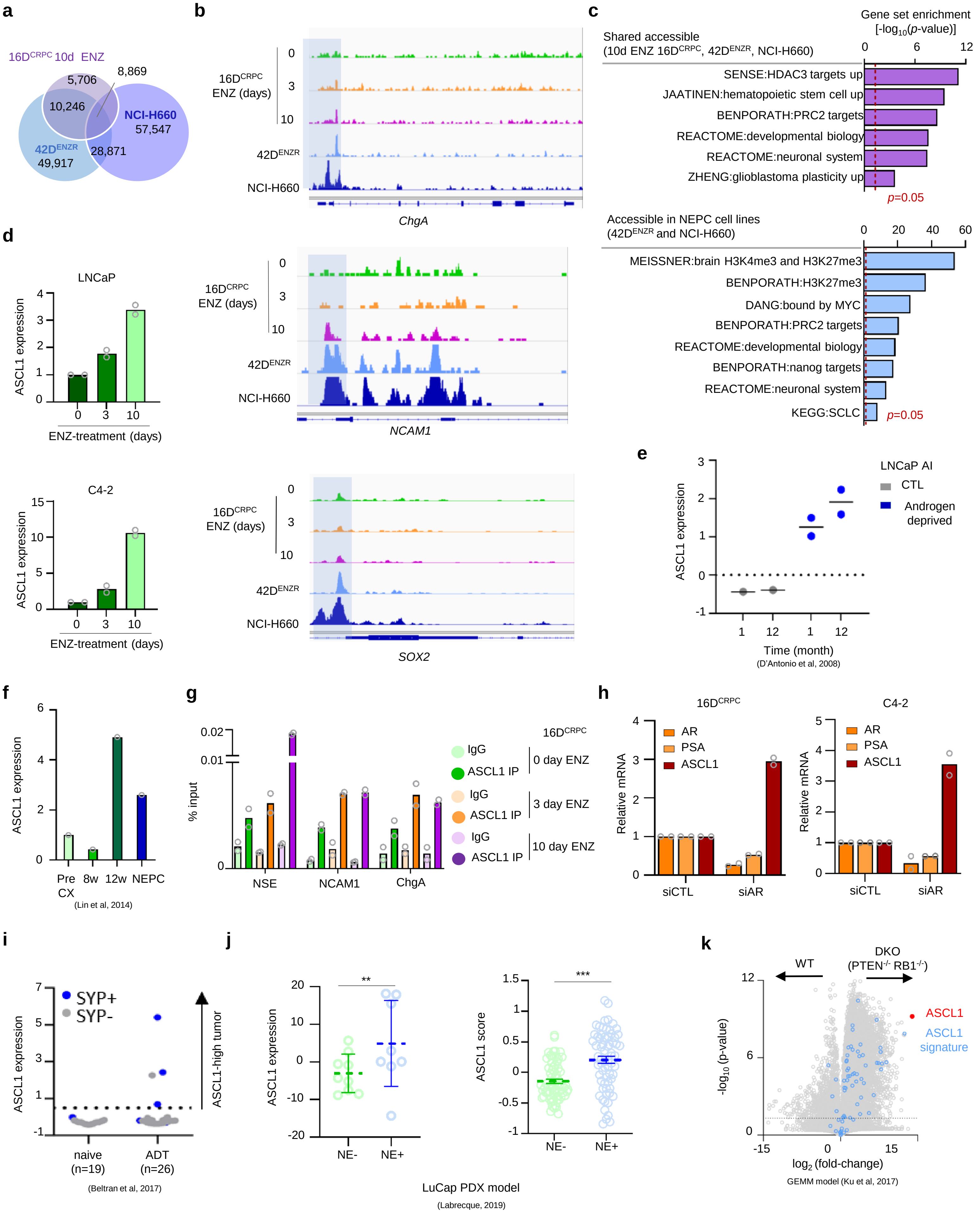

Supplemental Figure 2 – see next page for caption

Supplemental Figure 2

a) Venn diagram shows chromatin accessibility distribution. Data presented as number of regions.

b) Epigenomic alterations shows chromatin accessibility in genetic loci of *CHGA*, *NCAM1* and *SOX2* across ENZ-treated 16D^{CRPC} and neuroendocrine prostate cancer (NEPC). Data visualized using IGV .

c) Pathways associated with shared accessible (top) and NEPC regions (bottom). Dotted line represents p=0.05, statistical analysis was performed using a hypergeometric test.

d) Relative mRNA normalized to GAPDH (n=2 biologically independent replicates).

e) ASCL1 expression reported as log2FoldChange[75].

f) Relative ASCL1 mRNA expression normalized to pre-castration (pre CX) in LT331 model[19].

g) ChIP-PCR shows temporal increase of ASCL1 binding at neuroendocrine genes *CHGA*, *NCAM1* and *NSE*, following ASCL1 over-expression in castration-resistant prostate cancer (CRPC) (0 day), which was enhanced when combined with CSS+ENZ over a time course of 3 and 10 days (n=2 biologically independent samples). Data visualized using IGV.

h) CRPC siAR presented as relative mRNA normalized to GAPDH (n=2 biologically independent samples).

i) High ASCL1 expressing tumors exhibit high SYP expression. Data reported as z-score[15].

j) ASCL1 expression reported as log2FPKM mean±SD, *p*=0.0058, NE- n=9 and Ne+ n=8 biologically independent animals (left) ASCL1 activity reported as z-score, mean±SEM, *p*=0.0004 (right) in LuCaP PDX[12]. Two-tailed unpaired t-test.

k) ASCL1 expression and activity in DKO vs wild type (WT) GEMMs[14]. Each dot represents a gene, with “ASCL1 signature” (blue) and “ASCL1” (red) highlighted. Dotted line represent *p*-value=0.05. Statistical analysis was performed using a cumulative hypergeometric test.

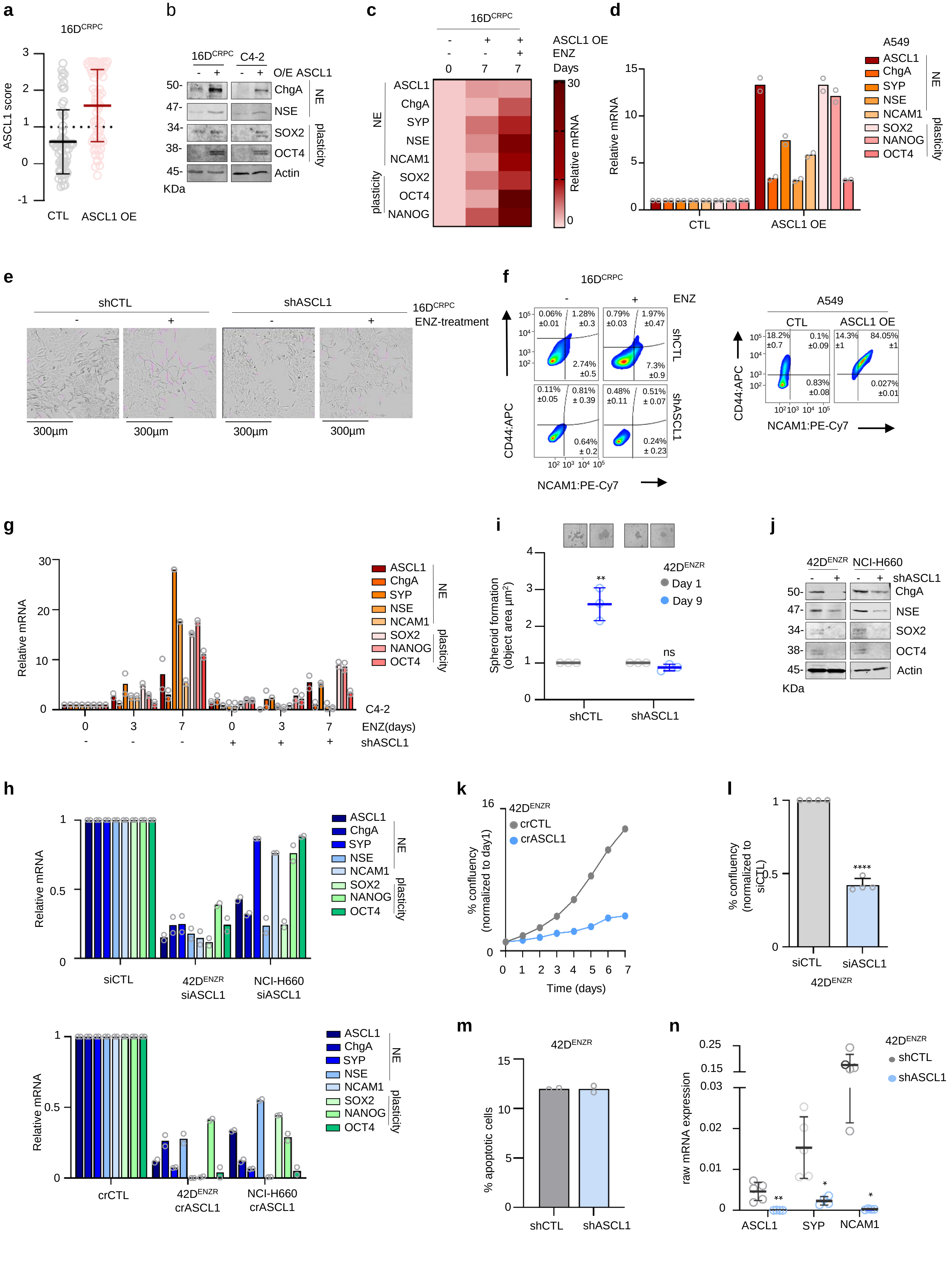

Supplemental Figure 3 – see next page for caption

Supplemental Figure 3

a) ASCL1 score reported as z-score mean ± SD. (16D^{CRPC} CTL n=2 and ASCL1 OE n=1 biologically independent samples).

b) Western blot shows expression of neuronal (NE) and cancer stem cell (CSC) markers in CRPC overexpressing ASCL1, actin was used as loading control (n=3 biologically independent samples).

c) ASCL1 overexpression in combination with ENZ (n=2 biologically independent samples).

d) NE and CSC (up) NCAM1 and CD44 (down) in lung adenocarcinoma cell line A549 overexpressing ASCL1 (n=2 biologically independent samples).

e) 7-days post-ENZ, “neurite-like extensions” (pink), scale bar=300μm (n=3 biologically independent samples).

f) NCAM1 and CD44 in 16D^{CRPC} shASCL1 7 days post-ENZ (n=2 biologically independent samples).

g) C4-2 shASCL1 treated with ENZ (n=2 independent samples).

h) NE and CSC in NEPC siASCL1 (top) and CRISPR ASCL1 (bottom) (n=2 independent samples).

i) Spheroid formation reported as mean ± SD (CTL p=0.0032 and shASCL1 p=0.0819, two-tailed unpaired t-test, n=3 biologically independent samples).

j) Western blot shows expression of NE and CSC in markers in NEPC shASCL1, actin was used as loading control (n=3 biologically independent samples).

k) 42D^{ENZR} CRISPR ASCL1 proliferation (n=2 biologically independent samples).

l) 42D^{ENZR} siASCL1 proliferation at day 7 reported as mean±SD, with p-value calculated at the end-point, p<0.0001; two-tailed unpaired t-test (n=4 biologically independent samples).

m) Apoptosis reported as percentage of apoptotic cell (n=2 biologically independent samples).

n) NE mRNA expression in tumors. Each dot represents a mice. Data reported as mean±SD, ASCL1 p=0.0046, SYP p=0.0113, and NCAM1 p=0.0421; two-tailed unpaired t-test (shCTL n=5 and shASCL1 n=4 biologically independent animal).

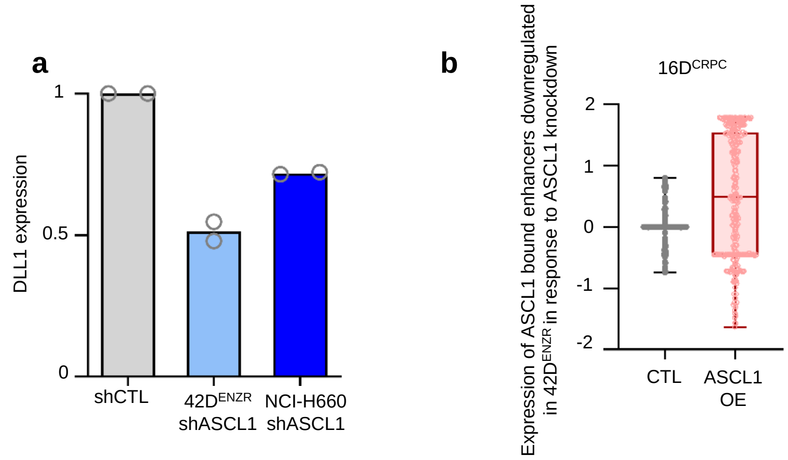

Supplemental Figure 4

a) Relative mRNA expression of DLL1 after knockdown of ASCL1 in 42D^{ENZR} and NCI-H660, normalized to GAPDH, presented as (n=2 biologically independent samples).

b) Increase expression of enhancers (bound by ASCL1 and identified to be downregulated following knockdown of ASCL1) in 16D^{CRPC} following over-expression of ASCL1. Data reported as z-score. Box plot shows median as central line, bounds of the box represents quartiles, and whiskers show minimum and maximum value with each dot represent one gene (16D^{CRPC} CTL n=2 and ASCL1 OE n=1 biologically independent sample).

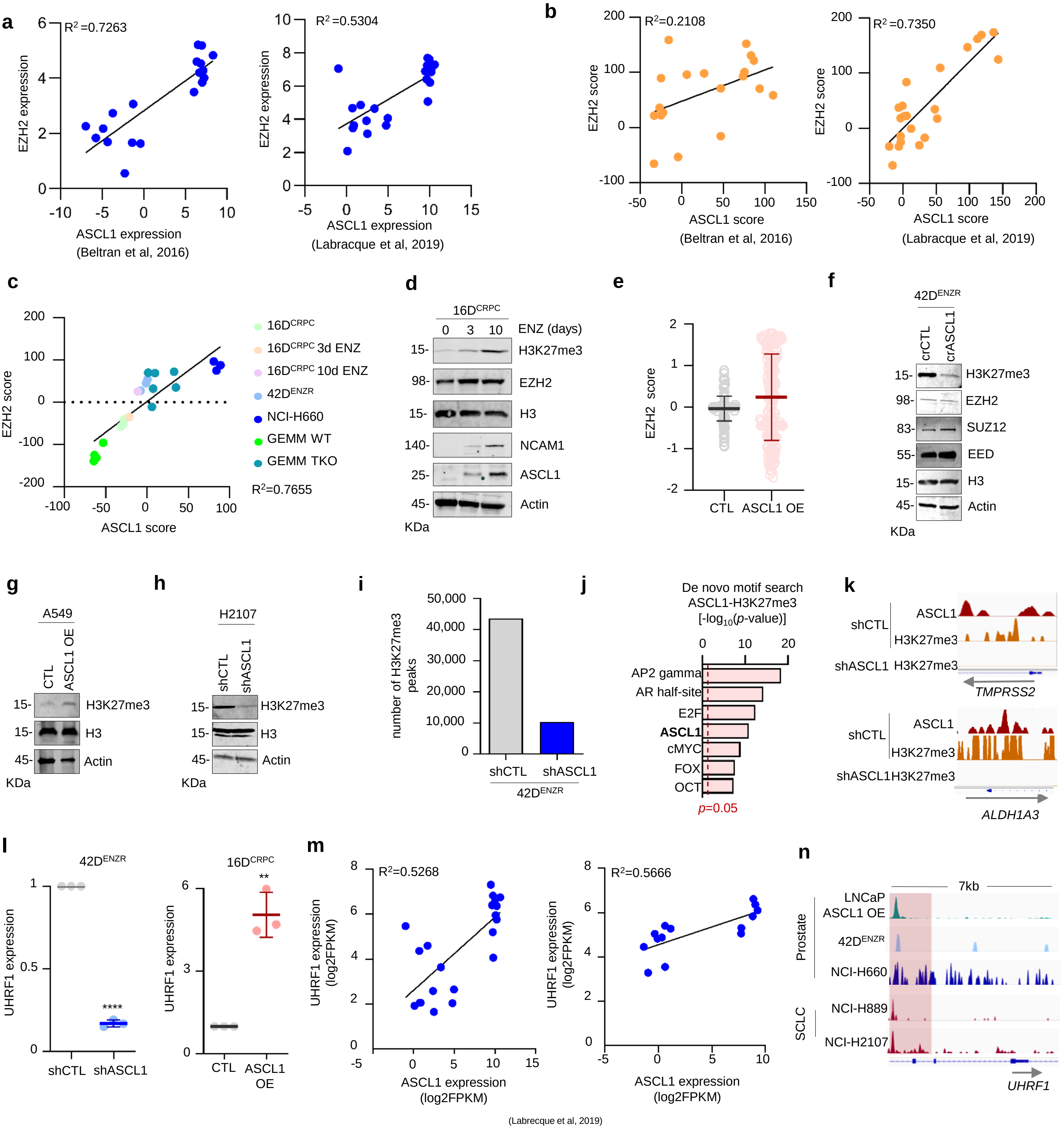

Supplemental Figure 5 – see next page for caption

Supplemental Figure 5

a) Correlation between ASCL1 and EZH2 expression reported as z-score in Beltran[15] (right) and Labrecque[12] (left) cohort (R^2=0.7263 and R^2=0.5304).

b) Correlation between ASCL1 and EZH2 custom score reported as z-score in Labrecque[12] (right) and Beltran[15] (left) cohort (R^2=0.2108 and R^2=0.7350).

c) Correlation between ASCL1 and EZH2 in prostate cancer cell lines.

d) Western blot shows expression of ASCL1 and EZH2 and their target genes in $16D^{CRPC}$ following ENZ treatment, actin and H3 were used as loading controls (n=3 biologically independent samples).

e) EZH2 score in $16D^{CRPC}$ control and over-expression ASCL1 reported as z-score normalization of each gene, mean±SD, CTL=2 and OE=1 biologically independent samples).

f) Western blot shows Polycomb Repressive complex 2 (PRC2) subunits expression and activity as measured by H3K27me3 in $42D^{ENZR}$ following crASCL1, actin and H3 were used as loading controls (n=3 biologically independent samples).

g) Western blot shows H3K27me3 expression in lung cancer adenocarcinoma (Adeno), A549 over-expressing ASCL1, actin was used as loading control (n=3 biologically independent samples).

h) Western blot shows H3K27me3 expression in small-cell lung cancer (SCLC), H2107 shASCL1, actin was used as loading control (n=3 biologically independent samples).

i) Number of total H3K27me3 peaks in $42D^{ENZR}$ shCTL and shASCL1 generated from ChIPseq (n=1 sample).

j) ASCL1-H3K27me3 co-bound regions de-novo motif search. Statistical analysis was performed using a hypergeometric test.

k) Genomic loci shows relative occupation over input of ASCL1 and H3K27me3 at AR/luminal genes (*TAMPRSS2* and *ALDH1A3*) and loss of H3K27me3 at these regions in $42D^{ENZR}$ shASCL1 using IGV.

l) Expression of UHRF1 reported as log2FPKM mean±SD, $42D^{ENZR}$ p<0.0001 and $16D^{CRPC}$ p=0.0012; two-tailed unpaired t-test (n=3 biologically independent samples).

m) Correlation between expression of ASCL1 and UHRF1 in Labrecque NEPC patients[12] (left) and in NE positive LuCaP PDX[12] (right) (R^2=0.5268 and R^2=5666) presented as log2FPKM.

n) ChIPseq of ASCL1 shows binding to enhancer of UHRF1 in prostate and SCLC using IGV.

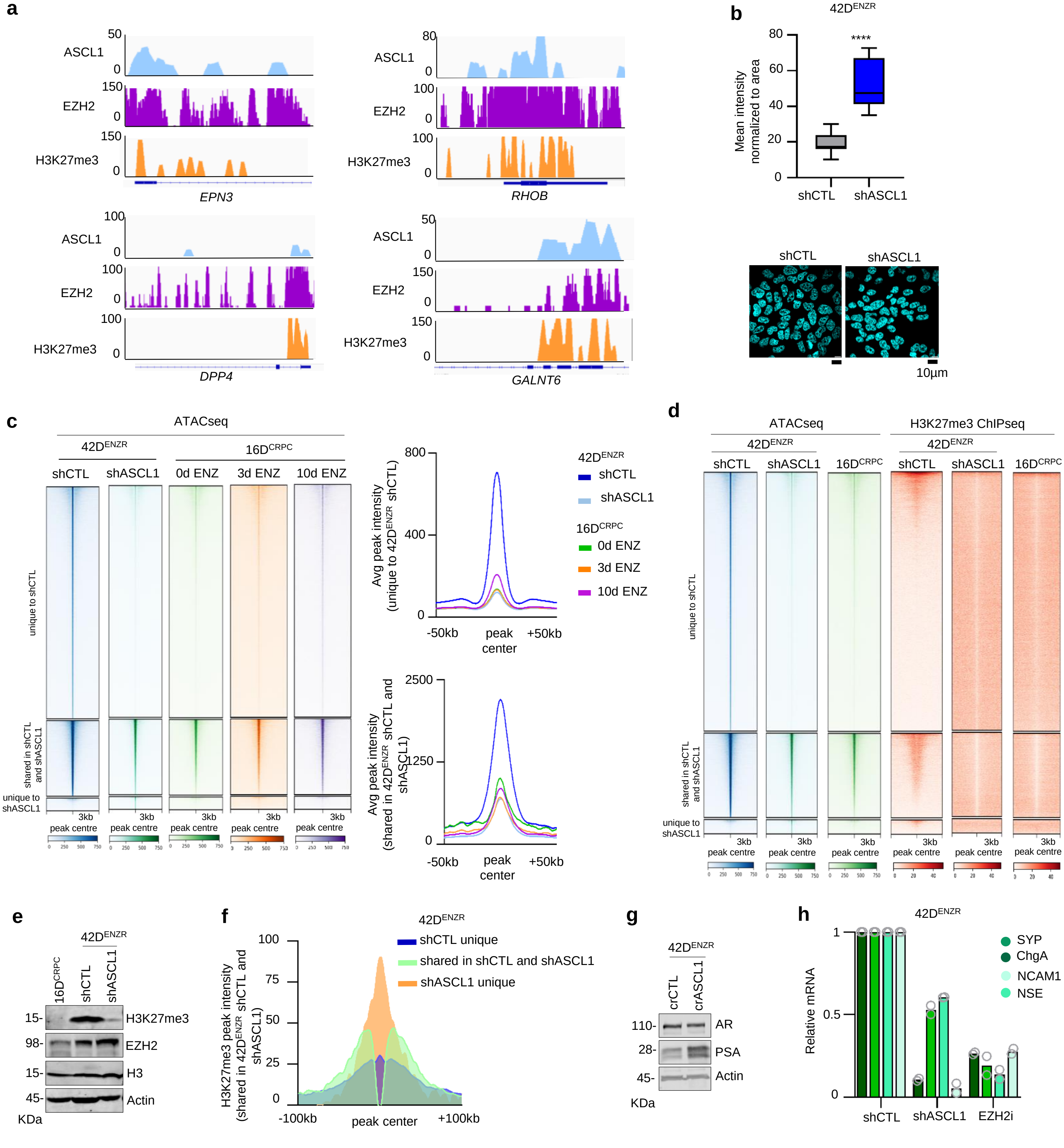

Supplemental Figure 6

a) Luminal genes co-bound by ASCL1 and EZH2 and are marked with H3K27me3 visualized using IGV.

b) Hoechst staining shows chromatin condensation in $42D^{ENZR}$ shASCL1. scale bar=10µm . Box plot shows median as central line, bounds of the box represents quartiles, and whiskers show minimum and maximum value, $p<0.0001$, two-tailed unpaired t-test, n=15 biologically independent cells).

c) Heatmap of chromatin accessible in $42D^{ENZR}$ shCTL and shASCL1; $16D^{CRPC}$ control and ENZ treatment, presented as 3kb window around the peak center (left) average accessible peak intensity (right).

d) Overlaying of H3K27me3 ChIPseq shown as average intensity over input (orange) with ATACseq.

e) Western blot shows H3K27me3 expression in CRPC compare to $42D^{ENZR}$ shCTL and shASCL1, with H3 and actin as loading controls (n=3 biologically independent samples).

f) Histogram shows different pattern of H3K27me3 in unique accessible regions in control $42D^{ENZR}$ or shared from ChIPseq.

g) Expression of prostate specific antigen (PSA) following CRISPR knockout of ASCL1 in $42D^{ENZR}$ with actin as loading control (n=3 biologically independent samples).

h) Down-regulation of NE markers in $42D^{ENZR}$ with inhibition of EZH2 (EZH2i) or shASCL1. mRNA expression normalized to GAPDH (n=2 biologically independent samples).

Supplemental Figure 7. Flow Cytometry Gating. Flow cytometry gating strategy used in Fig. 3E and Supplementary Data Fig. 3D and 3F.

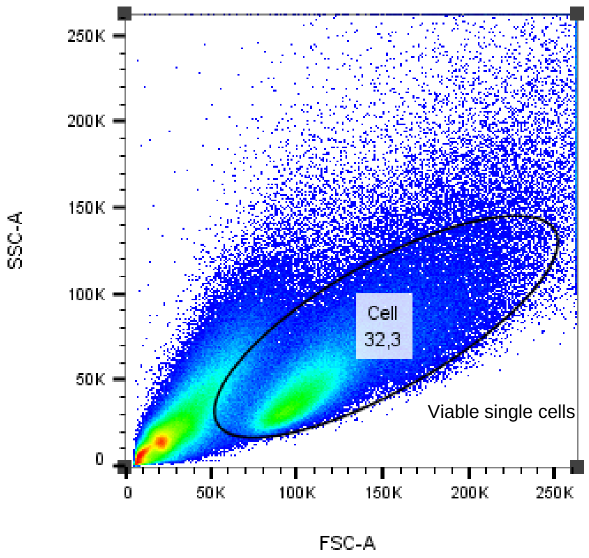

Supplemental Figure 3

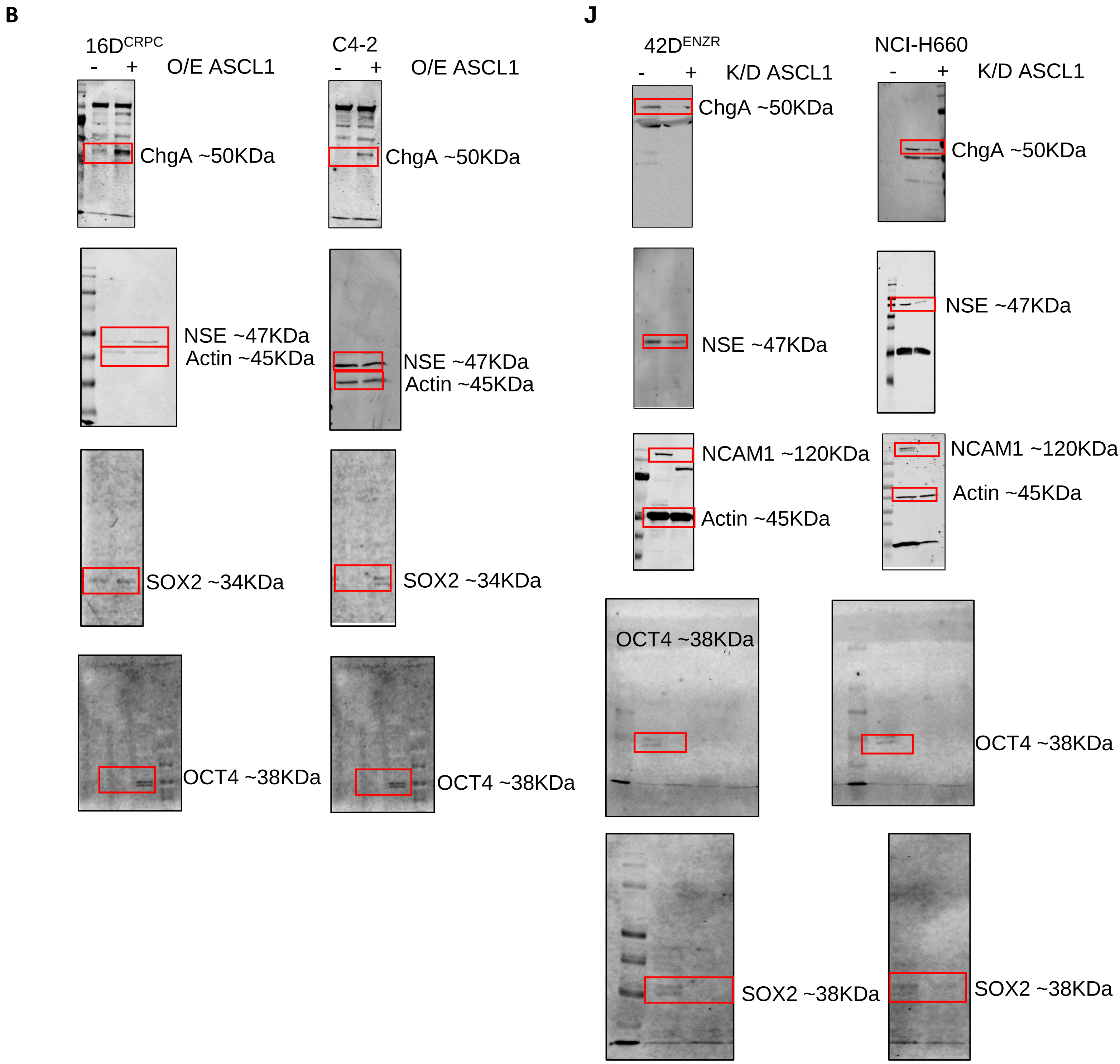

Supplemental Figure 5

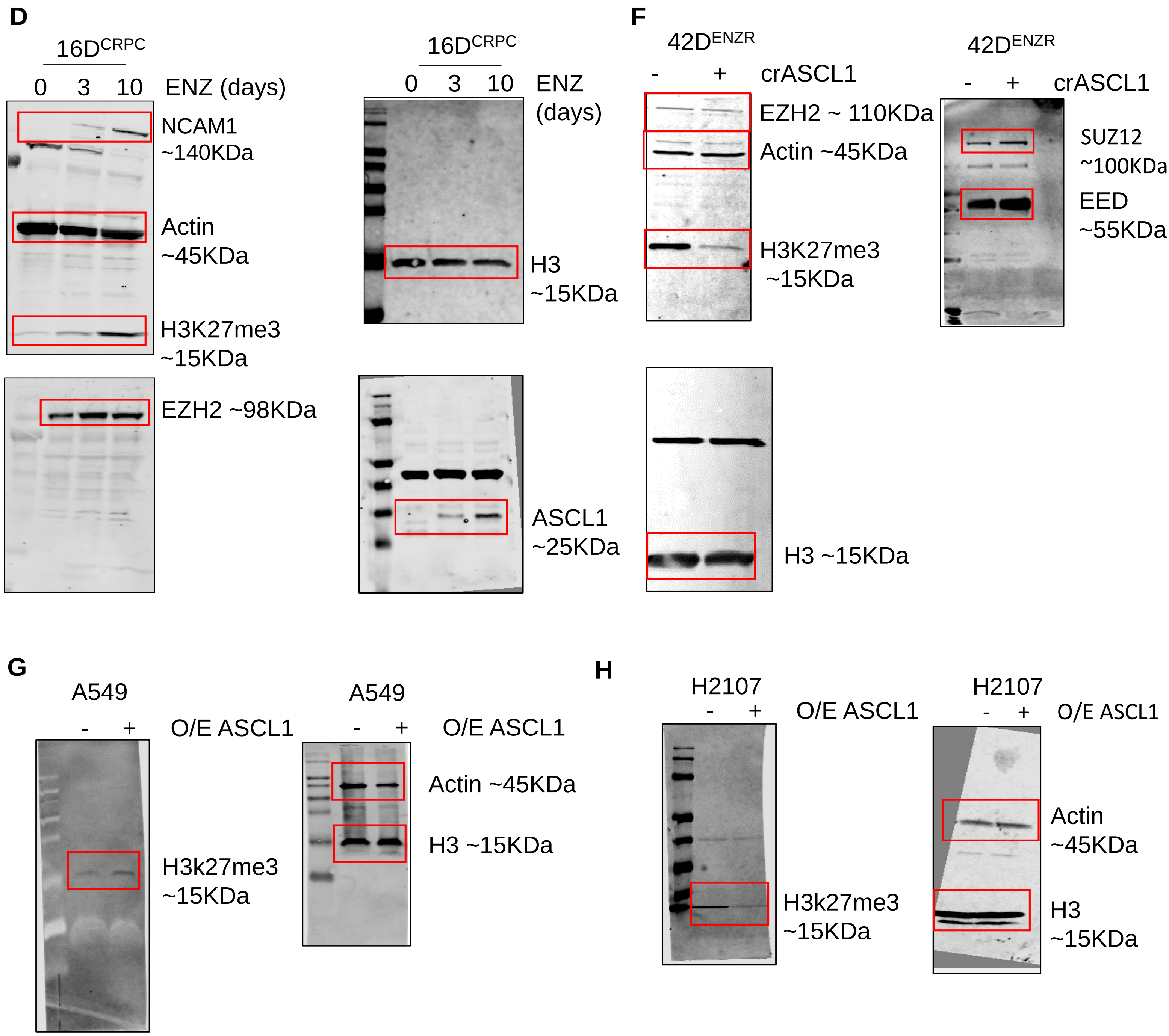

Supplemental Figure 6

E

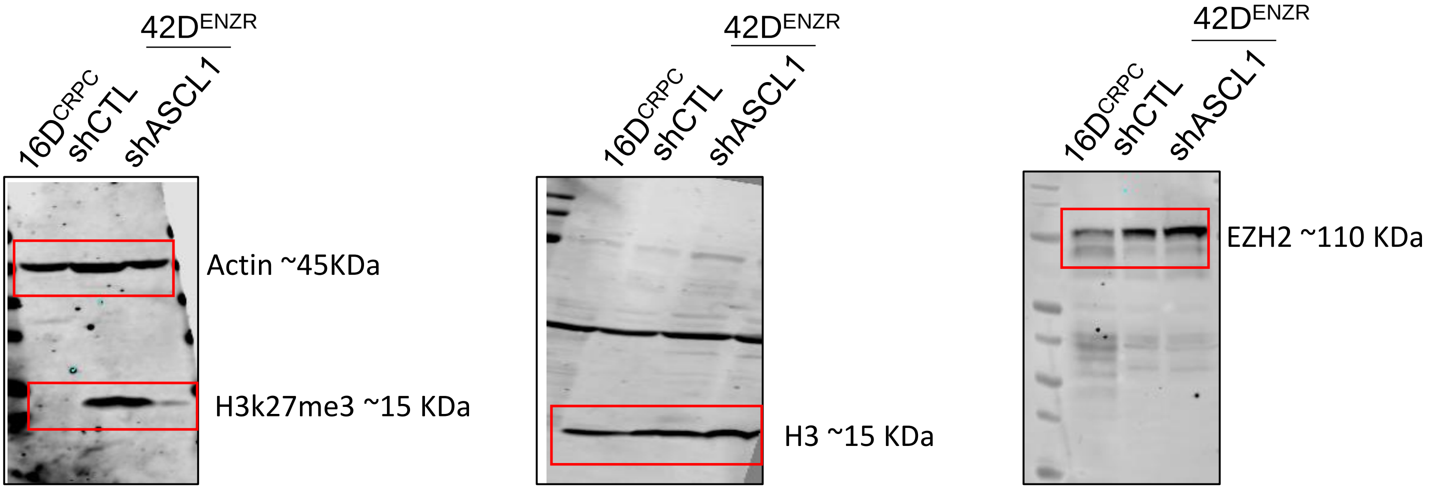

G

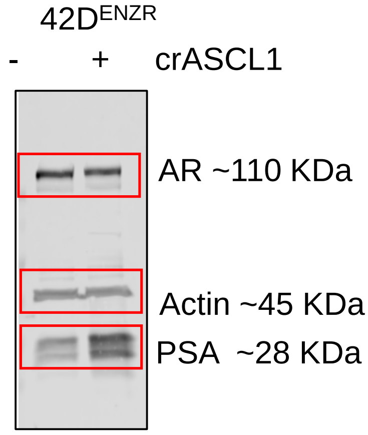

Supplement: Supplementary file 1 — Supplementary Information [file 41467_2022_29963_MOESM1_ESM.pdf]
