# Supplementary material for: ASCL1 activates neuronal stem cell-like lineage programming through remodeling of the chromatin landscape in prostate cancer
Source: Nat Commun. 2022 Apr 27;13:2282. doi: 10.1038/s41467-022-29963-5 (PMC9046280; doi:10.1038/s41467-022-29963-5)
Supplement: Supplementary file 2 — Description of Additional Supplementary Files [file 41467_2022_29963_MOESM2_ESM.pdf]

Description of Additional Supplementary Files

File Name: Supplementary Data 1

Description: List of abbreviations

File Name: Supplementary Data 2

Description: Ranked TF motifs surrounding accessible peak in 16DCRPC ENZ-treated.

File Name: Supplementary Data 3

Description: Genes annotated to ASCL1 motif in accessible regions unique to 10 days ENZ-treated $16D^{CRPC}$

File Name: Supplementary Data 4

Description: Ranked TF motifs surrounding accessible peak in CPRC ENZ-treated vs NEPC
