# Supplementary material for: ASCL1 activates neuronal stem cell-like lineage programming through remodeling of the chromatin landscape in prostate cancer
Source: Nat Commun. 2022 Apr 27;13:2282. doi: 10.1038/s41467-022-29963-5 (PMC9046280; doi:10.1038/s41467-022-29963-5)
Supplement: Supplementary file 7 — Reporting Summary [file 41467_2022_29963_MOESM7_ESM.pdf]

nature portfolio

Corresponding author(s): Amina Zoubeidi

Last updated by author(s): Apr 7, 2022

# Reporting Summary

Nature Portfolio wishes to improve the reproducibility of the work that we publish. This form provides structure for consistency and transparency in reporting. For further information on Nature Portfolio policies, see our Editorial Policies and the Editorial Policy Checklist.

## Statistics

For all statistical analyses, confirm that the following items are present in the figure legend, table legend, main text, or Methods section.

| n/a | Confirmed | |
|---|---|---|
| [ ] | [x] | The exact sample size (*n*) for each experimental group/condition, given as a discrete number and unit of measurement |
| [ ] | [x] | A statement on whether measurements were taken from distinct samples or whether the same sample was measured repeatedly |
| [ ] | [x] | The statistical test(s) used AND whether they are one- or two-sided<br>*Only common tests should be described solely by name; describe more complex techniques in the Methods section.* |
| [x] | [ ] | A description of all covariates tested |
| [x] | [ ] | A description of any assumptions or corrections, such as tests of normality and adjustment for multiple comparisons |
| [ ] | [x] | A full description of the statistical parameters including central tendency (e.g. means) or other basic estimates (e.g. regression coefficient) AND variation (e.g. standard deviation) or associated estimates of uncertainty (e.g. confidence intervals) |
| [ ] | [x] | For null hypothesis testing, the test statistic (e.g. *F*, *t*, *r*) with confidence intervals, effect sizes, degrees of freedom and *P* value noted<br>*Give P values as exact values whenever suitable.* |
| [x] | [ ] | For Bayesian analysis, information on the choice of priors and Markov chain Monte Carlo settings |
| [x] | [ ] | For hierarchical and complex designs, identification of the appropriate level for tests and full reporting of outcomes |
| [x] | [ ] | Estimates of effect sizes (e.g. Cohen's *d*, Pearson's *r*), indicating how they were calculated |

*Our web collection on statistics for biologists contains articles on many of the points above.*

## Software and code

Policy information about availability of computer code

| | |
|---|---|
| Data collection | ChIP-seq and RNA-seq were performed on Illumina NextSeq 500 sequencer.<br>ATAC-seq were performed on Illumina HiSeq 2500 and NOVAseq 6000 sequencer.<br>Western blots were acquired using the LICOR Odyssey Imaging System.<br>Flow cytometry was performed using a BD FACSCanto II Cell Analyzer with FlowJo software (version 10.4.2).<br>Real-time PCR was performed using a ABI ViiA7.<br>Cell growth was acquired using an IncuCyte S3.<br>Fluorescent images were taken using FV3000RS confocal microscope equipment using Olympus software FV31S-SW version 2.3.2.169<br>Chromatin condensation quantification was used using ImageJ (version 1.8.0) |
| Data analysis | FastQC (v0.11.9) for checking raw NGS data quality.<br>BWA-MEM software (v0.7.17) with default parameters for mapping and alignment of ChIP-seq and ATAC-seq data.<br>Custom AWK code was used for quality filtering of reads (egrep -w 'NM:i:[0-5]\|@SQ\|@PG' fileName.sam \| awk '($5 == 60 && $2 < 2048) \|\| $1~/^@/' > fileName-Q60.sam).<br>Samtools software (v1.15) for generation of bam files.<br>MACS2 (v2.2.7.1) for peak calling with FDR q-value 0.05.<br>deepTools (v3.5.1) for heatmap visualization.<br>Bedtools (v2.30.0) program suite was used to generate shared and unique peaks.<br>HOMER (v4.11) for motif analysis.<br>Custom AWK code was used for parsing of annotated peak file (cat inputFileName.txt \| awk 'BEGIN{FS=OFS="\t"} { if(length($22)>2 && $3!="Start") print $2,$3,$4,$5,$6}' > outputFileName.bed)<br>RUV (v1.26.0) for batch correction of ATAC-seq samples. |

DiffBind (v3.2.7) was used for correlation analysis of ATAC-seq samples.
Cufflinks (v. 2.2.1) for RNA-seq.
Gene Set Enrichment Analysis (GSEA, v4.2.2) with pathways from the Molecular Signatures Database (v. 7.1) for pathway analysis.
Single sample GSEA (ssGSEA) was carried out using gProfiler web server
Li-Cor Image Studio (v. 4.2) for Western blots.
FlowJo (v. 10.4.2) for flow cytometry plots.
IncuCyte S3 software (v. 2018B) for cell proliferation and NeuroTrack.
GraphPad Prism (v. 7/8) for graphs and statistical analysis.
Olympus software FV31S-SW version 2.3.2.169 for fluorescent imaging.
Principal Component Analysis (PCA) was performed using the prcomp() function in R.
ImageJ (version 1.8.0) for chromatin condensation quantification.

https://github.com/ZoubeidiLab/Qualityfilter_SAM/commit/f7bfcbb9d2525212c7190317ddb58db471e0b56a
was used for quality filtering of ChIPseq and ATACseq reads as described in the methods section "ChIPseq and ATACseq data analysis". AWK code (cat inputFileName.txt | awk 'BEGIN{FS=OFS="\t"} { if(length($22)>2 && $3!="Start") print $2,$3,$4,$5,$6}' > outputFileName.bed) was used for parsing annotated peak file as described in methods section "Motif analysis".

For manuscripts utilizing custom algorithms or software that are central to the research but not yet described in published literature, software must be made available to editors and reviewers. We strongly encourage code deposition in a community repository (e.g. GitHub). See the Nature Portfolio guidelines for submitting code & software for further information.

## Data

Policy information about availability of data

All manuscripts must include a data availability statement. This statement should provide the following information, where applicable:
- Accession codes, unique identifiers, or web links for publicly available datasets
- A description of any restrictions on data availability
- For clinical datasets or third party data, please ensure that the statement adheres to our policy

RNA-seq, ChIP-seq, and ATAC-seq data generated in this study have been deposited and are available in the GEO database under the accession GSE183200 [https://www.ncbi.nlm.nih.gov/geo/query/acc.cgi?acc=GSE183200]. Publicly available gene expression data from the SU2C/PCF-West Coast Dream Team cohort was downloaded from Aggarwal et al (PMID:29985747) [https://pubmed.ncbi.nlm.nih.gov/29985747/], the Beltran 2016 cohort from Beltran et al (PMID:26855148) [https://pubmed.ncbi.nlm.nih.gov/26855148/], the CALGB 90203 cohort from Beltran et al (PMID:28842510) [https://pubmed.ncbi.nlm.nih.gov/28842510/], and the Labrecque et al (PMID:31361600) [https://pubmed.ncbi.nlm.nih.gov/31361600/]. The gene expression data from SKO/DKO/TKO prostate cancer GEMM was accessed through GEO:GSE90891[https://www.ncbi.nlm.nih.gov/geo/query/acc.cgi?acc=GSE90891], the LuCaP PDX was downloaded from GEO:GSE126078 [https://www.ncbi.nlm.nih.gov/geo/query/acc.cgi?acc=GSE126078], LT331 downloaded from GEO:GSE41193 [https://www.ncbi.nlm.nih.gov/geo/query/acc.cgi?acc=GSE41193], LNCaP cells with androgen deprivation downloaded from GEO:GSE8702 [https://www.ncbi.nlm.nih.gov/geo/query/acc.cgi?acc=GSE8702], and 16DCRPC and 42DENZR GSK126 RNAseq, 42D_EZH2IP, 42D_H3K27me3IP and SKO/DKO/TKO GEMM ATAC-seq was downloaded from GEO:GSE138460 [https://www.ncbi.nlm.nih.gov/geo/query/acc.cgi?acc=GSE138460]. LNCaP_ASCL1OE_ASCL1 ChIP-seq data was borrowed from Baca et al (PMID:33785741) [https://pubmed.ncbi.nlm.nih.gov/33785741/]. Transformed prostate and lung epithelial ATAC-seq data published in Park et al (PMID:30287662) was downloaded from GEO:GSE118204 [https://www.ncbi.nlm.nih.gov/geo/query/acc.cgi?acc=GSE118204]. LuCaP PDX ATAC-seq data published by Cejas et al (PMID: 34599169) was downloaded from GEO:GSE156291 [https://www.ncbi.nlm.nih.gov/geo/query/acc.cgi?acc=GSE156291].

# Field-specific reporting

Please select the one below that is the best fit for your research. If you are not sure, read the appropriate sections before making your selection.

☒ Life sciences ☐ Behavioural & social sciences ☐ Ecological, evolutionary & environmental sciences

For a reference copy of the document with all sections, see nature.com/documents/nr-reporting-summary-flat.pdf

# Life sciences study design

All studies must disclose on these points even when the disclosure is negative.

| | |
|---|---|
| Sample size | Sample sizes were determined based on estimates from preliminary experiments and similar studies so that reasonable statistical analysis could be conducted. For in vivo experiments, the number of mice assigned to each arm was selected to provide sufficient statistical power to discern significant differences, based on previous experience with the models used (Davies et al, Nature Cell Biology, 2021; Bishop et al, Cancer Discovery, 2017; Kuruma et al, Molecular Cancer Therapeutics, 2013). |
| Data exclusions | No data was excluded from our study. |
| Replication | All experiments were repeated at least 2 independent times and the replicating experiments produced similar results. |
| Randomization | Samples were randomly allocated. |
| Blinding | For in vivo studies, mice were inoculated/treated by one researcher and a separate research took tumour measurements to ensure the experiments were run in a blinded manner. Blinding was not performed for in vitro experiments as the individual performing the analysis was also involved in collecting and labeling the samples. However, all analysis within next-generation sequencing experiments were run at the |

same time in an automated manner, so prior knowledge of groups had no impact on data output.

# Reporting for specific materials, systems and methods

We require information from authors about some types of materials, experimental systems and methods used in many studies. Here, indicate whether each material, system or method listed is relevant to your study. If you are not sure if a list item applies to your research, read the appropriate section before selecting a response.

## Materials & experimental systems

| n/a | Involved in the study |
|---|---|
| ☐ | ☒ Antibodies |
| ☐ | ☒ Eukaryotic cell lines |
| ☒ | ☐ Palaeontology and archaeology |
| ☐ | ☒ Animals and other organisms |
| ☒ | ☐ Human research participants |
| ☒ | ☐ Clinical data |
| ☒ | ☐ Dual use research of concern |

## Methods

| n/a | Involved in the study |
|---|---|
| ☐ | ☒ ChIP-seq |
| ☐ | ☒ Flow cytometry |
| ☒ | ☐ MRI-based neuroimaging |

## Antibodies

Antibodies used

AR (Cell Signaling #5153; clone D6F11, 1:1000)
AMPK (Cell signaling #2532S, 1:500)
p-AMPK-T172 (Cell signaling #2535S, clone 40H9, 1:1000)
ASCL1 (Santa Cruz #sc-374104; clone D7,1:200)
ChgA(Abcam, #ab151610, 1:1000)
EED (Millipore #17-10034, 1:1000)
EZH2 (Active Motif #39933, 1:2000)
p-EZH2-T311 (Cell signaling, #27888S, 1:1000)
H3 (Cell signaling, #14269s, clone 1B1B2, 1:2000)
H3K27Me3 (Millipore #07-449, 1:2000)
NSE/ENO2 (Agilent #M0873, 1:1000)
OCT4 (Cell signaling D705Z, 7546S, 1:500)
PSA (Cell Signaling #5365 clone D6B1,1:5000)
SOX2 (Invitrogen, MA1-014, 1:500),
SUZ12 (Cell Signaling #3737S; clone D39F6, 1:1000),
UHRF1 (Cell signaling, #12387S, 1:1000)
β-actin (Sigma #A2228;clone AC-74, 1:25,000)
IRDye 800CW donkey anti-Rabbit (LI-COR, 926-32213, 1:10,000)
IRDye 680CW donkey anti-mouse (10,000, LI-COR, 926-68072, 1:10,000) were used as secondary antibodies.

The following antibodies were used for ChIP-seq: ASCL1 (10µg, Abcam, cat #556604), EZH2 (5µg, Active Motif, cat #39933), H3K27Me3 (5µg; Millipore, cat #07-449).

The following antibodies were used for flow cytometry: CD56/NCAM1, PE-Cyanine7-conjugated (Clone CMSSB; 1:40, Thermo #25-0567-42, Lot #2100317) and CD44, APC-conjugated (Clone IM7; 1:40, Thermo #17-0441-82, Lot #2023951).

The following antibodies were used for immunofluorescence: EZH2 (active motif, cat #3993, 1:20), Alexa fluor 488 (Invitrogen, cat. #A21206, 1:10,000), DAPI (Thermo, cat. #:D1306, 1:500).

Validation

All antibodies used in this study were commercial and validated by the manufactures in human samples.
For example the AR (Cell signaling #5153) has been validated by the manufacturer using SimpleChIP® Enzymatic Chromatin IP Kits.
EED (Millipore #17-10034) was validated by the manufacturer using chromatin precipitation.

## Eukaryotic cell lines

Policy information about cell lines

Cell line source(s)

NCI -H660 (cat. #CRL-5813), C4-2 (cat. #CRL-3314), A549 (cat. #CRM-CCL-185) and H2107 (cat. #CRL-5983_FL) cell lines were obtained from ATCC. HEK293T (cat. #R70007) were obtained from ThermoFisher. CRPC (16DCRPC) and ENZ-resistant AR+ NE-like (42DENZR) cell lines were generated from LNCaP cells, previously detailed by our group Bishop et al (PMID: 27784708) and Davies et al (PMID:34489572). We utilized an in vivo model of CRPC and ENZ resistance previously developed by us 54-56 that mirrors clinically reported treatment refractory phenotypes. LNCaP cells were inoculated into mice and upon castration CRPC tumors (16DCRPC) emerged. Further treatment of 16DCRPC tumors with ENZ (10mg/kd/d) lead to the re-emergence of tumors with heterogeneous resistance mechanisms, including lineage plasticity. 42DENZR tumors exhibit AR expression, but loss of canonical AR signaling, concomitant with an enrichment in plasticity and neuronal transcriptional

| | |
|---|---|
| | programs. |
| Authentication | All cell lines have been authenticated via STR profiling. |
| Mycoplasma contamination | All cell lines tested negative for mycoplasma in routine checks using the Lonza MycoAlert kit. |
| Commonly misidentified lines (See ICLAC register) | No commonly misidentified cell lines were used in the study. |

# Animals and other organisms

Policy information about studies involving animals; ARRIVE guidelines recommended for reporting animal research

| | |
|---|---|
| Laboratory animals | Mouse, strain: NU-Foxn1nu, 6-8 weeks old, male (Envigo). Housing conditions: 4/cage; Light/dark cycle: 12h; Ambient temperature: 21-22C; Humidity: 25-47%. |
| Wild animals | This study did not involve wild animals. |
| Field-collected samples | This study did not involve samples collected from the field. |
| Ethics oversight | All animal studies were performed in accordance with protocols approved by the Animal Care Committee at the University of British Columbia (A16-0246). |

Note that full information on the approval of the study protocol must also be provided in the manuscript.

# ChIP-seq

## Data deposition

☒ Confirm that both raw and final processed data have been deposited in a public database such as GEO.

☒ Confirm that you have deposited or provided access to graph files (e.g. BED files) for the called peaks.

Data access links
*May remain private before publication.*

GEO:GSE183200

Files in database submission

Raw sequencing files (fastq) and coverage files (bigwig) are deposited for ChIP-seq and ATAC-seq experiments. Raw sequencing files (fastq) and read counts (text) are deposited for RNA-seq data.

ChIP-seq
16D-CRPC-H3K27me3-ChIP.fastq.gz
16D-CRPC-H3K27me3-ChIP.bw
16D-CRPC-H3K27me3-ChIP-broad_peak.broadPeak
16D-CRPC-Input.fastq.gz
42D-ENZR-ASCL1-ChIP.fastq.gz
42D-ENZR-ASCL1-ChIP.bw
42D-ENZR-ASCL1-ChIP-peaks.narrowPeak
42D-ENZR-Input.fastq.gz
42D-ENZR-shASCL1-EZH2-ChIP.fastq.gz
42D-ENZR-shASCL1-EZH2-ChIP.bw
42D-ENZR-shASCL1-EZH2-ChIP-broad_peaks.broadPeak
42D-ENZR-shASCL1-H3K27me3-ChIP.fastq.gz
42D-ENZR-shASCL1-H3K27me3-ChIP.bw
42D-ENZR-shASCL1-H3K27me3-ChIP-broad_peaks.broadPeak
42D-ENZR-shASCL1-Input.fastq.gz
NCI-H660-ASCL1-ChIP-R1.fastq.gz
NCI-H660-ASCL1-ChIP-R2.fastq.gz
NCI-H660-Input-ChIP-R1.fastq.gz
NCI-H660-Input-ChIP-R2.fastq.gz
NCI-H660-ASCL1-ChIP.bw
NCI-H660-ASCL1-ChIP-peak.narrowPeak

ATAC-seq
16D-CRPC-0d-ENZ-R1-ATAC.fastq.gz
16D-CRPC-0d-ENZ-R2-ATAC.fastq.gz
16D-CRPC-0d-ENZ-AccDNA.bw
16D-CRPC-0d-ENZ-R2.1-ATAC.fastq.gz
16D-CRPC-0d-ENZ-R2.2-ATAC.fastq.gz
16D-CRPC-0d-ENZ-R3.1-ATAC.fastq.gz
16D-CRPC-0d-ENZ-R3.2-ATAC.fastq.gz
16D-CRPC-0d-ENZ-AccDNA-peak.narrowPeak
16D-CRPC-3d-ENZ-R1-ATAC.fastq.gz

16D-CRPC-3d-ENZ-R2-ATAC.fastq.gz
16D-CRPC-3d-ENZ-AccDNA.bw
16D-CRPC-3d-ENZ-R2.1-TAC.fastq.gz
16D-CRPC-3d-ENZ-R2.2-ATAC.fastq.gz
16D-CRPC-3d-ENZ-R3.1-ATAC.fastq.gz
16D-CRPC-3d-ENZ-R3.2-ATAC.fastq.gz
16D-CRPC-3d-ENZ-AccDNA-peak.narrowPeak
16D-CRPC-10d-ENZ-R1-ATAC.fastq.gz
16D-CRPC-10d-ENZ-R2-ATAC.fastq.gz
16D-CRPC-10d-ENZ-AccDNA.bw
16D-CRPC-10d-ENZ-R2.1-ATAC.fastq.gz
16D-CRPC-10d-ENZ-R2.2-ATAC.fastq.gz
16D-CRPC-10d-ENZ-R3.1-ATAC.fastq.gz
16D-CRPC-10d-ENZ-R3.2-ATAC.fastq.gz
16D-CRPC-10d-ENZ-AccDNA-peak.narrowPeak
42D-ENZR-R1-ATAC.fastq.gz
42D-ENZR-R2-ATAC.fastq.gz
42D-ENZR-AccDNA.bw
42D-ENZR-AccDNA-peak.narrowPeak
42D-ENZR-shASCL1-R1-ATAC.fastq.gz
42D-ENZR-shASCL1-R2-ATAC.fastq.gz
42D-ENZR-shASCL1-AccDNA.bw
42D-ENZR-shASCL1-AccDNA-peak.narrowPeak
NCI-H660-R1-ATAC.fastq.gz
NCI-H660-R2-ATAC.fastq.gz
NCI-H660-AccDNA.bw
NCI-H660-AccDNA-peak.narrowPeak

RNA-seq
CellLines_RNAseq_FPKM_Data.txt
16D-CRPC-3d-ENZ-RNAseq-R1.fastq.gz
16D-CRPC-3d-ENZ-RNAseq-R2.fastq.gz
16D-CRPC-10d-ENZ-RNAseq-R1.fastq.gz
16D-CRPC-10d-ENZ-RNAseq-R2.fastq.gz
16D-CRPC-ASCL1OE-RNAseq-R1.fastq.gz
16D-CRPC-ASCL1OE-RNAseq-R2.fastq.gz
16D-CRPC-RNAseq-R1.fastq.gz
16D-CRPC-RNAseq-R2.fastq.gz
42D-ENZR-Rep1-RNAseq-R1.fastq.gz
42D-ENZR-Rep1-RNAseq-R2.fastq.gz
42D-ENZR-Rep2-RNAseq-R1.fastq.gz
42D-ENZR-Rep2-RNAseq-R2.fastq.gz
42D-ENZR-Rep3-RNAseq-R1.fastq.gz
42D-ENZR-Rep3-RNAseq-R2.fastq.gz
42D-ENZR-shASCL1-Rep1-RNAseq-R1.fastq.gz
42D-ENZR-shASCL1-Rep1-RNAseq-R2.fastq.gz
42D-ENZR-shASCL1-Rep2-RNAseq-R1.fastq.gz
42D-ENZR-shASCL1-Rep2-RNAseq-R2.fastq.gz
42D-ENZR-shASCL1-Rep3-RNAseq-R1.fastq.gz
42D-ENZR-shASCL1-Rep3-RNAseq-R2.fastq.gz
NCI-H660-Rep1-RNAseq-R1.fastq.gz
NCI-H660-Rep1-RNAseq-R2.fastq.gz
NCI-H660-Rep2-RNAseq-R1.fastq.gz
NCI-H660-Rep2-RNAseq-R2.fastq.gz
NCI-H660-siASCL1-RNAseq-R1.fastq.gz
NCI-H660-siASCL1-RNAseq-R2.fastq.gz

Genome browser session
(e.g. UCSC)

We deposit and now available our data on GEO and we will not deposit on Genome Browser session

## Methodology

Replicates

ChIP-seq and ATAC-seq were done with one biological replicate.

Sequencing depth

ChIP-seq were 75bp single-end, except NCI-H660-ASCL1IP that was done as pair-end.

| sample name | Total reads | Uniquely mapped reads |
| --- | --- | --- |
| 42D-ENZR-ASCL1IP: | 11,037,432 | 2,319,874 |
| 42D-ENZR-Input: | 55,012,106 | 45,653,235 |

| | | |
|---|---|---|
| 42D-ENZR-H3K27me3IP: | 12,937,677 | 2,455,626 |
| 42D-ENZR-Input: | 50,298,119 | 34,595,021 |
| 42D-ENZR-shASCL1-H3k27me3IP: | 44,020,241 | 34,464,371 |
| 42D-ENZR-shASCL1-EZH2IP: | 16,523,273 | 12,690,926 |
| 42D-ENZR-shASCL1-Input: | 52,445,157 | 40,014,708 |
| NCI-H660-ASCL1IP: | 31,146,320 | 13,071,711 |
| NCI-H660-Input: | 84,354,007 | 56,252,937 |

ATAC-seq samples were 75bp or 125bp or 150bp paired-end.

| sample name | Total reads | Uniquely mapped reads |
|---|---|---|
| 16D-CRPC-0d-ENZ-R1: | 57,614,650 | 46,507,471 |
| 16D-CRPC-3d-ENZ-R1: | 57,016,082 | 46,948,606 |
| 16D-CRPC-10d-ENZ-R1: | 66,301,936 | 53,979,315 |
| 16D-CRPC-0d-ENZ-R2: | 48,043,616 | 39,162,119 |
| 16D-CRPC-3d-ENZ-R2: | 308,840,935 | 246,659,937 |
| 16D-CRPC-10d-ENZ-R2: | 223,794,423 | 179,763,279 |
| 16D-CRPC-0d-ENZ-R3: | 154,611,484 | 111,766,834 |
| 16D-CRPC-3d-ENZ-R3: | 268,282,352 | 210,946,123 |
| 16D-CRPC-10d-ENZ-R3: | 259,294,500 | 204,143,631 |
| 42D-ENZR: | 72,771,167 | 63,664,423 |
| 42D-ENZR-shASCL1: | 70,168,837 | 50,839,950 |
| NCI-H660: | 60,911,461 | 53,851,203 |

**Antibodies**

ASCL1 (5µg, Abcam, cat #556604), EZH2 (5µg, Active Motif, cat #39933), H3K27Me3 (5µg; Millipore, cat #07-449)

**Peak calling parameters**

For checking data quality using FastQC:
fastqc -o <outputDirName> fileName.fastq.gz

For mapping and alignment of fastq using default parameters:
bwa mem -t <n> ref.fa fileName.fastq > fileName.sam

samtools view with default parameters for converting sam to bam.
samtools sort with default parameters for sorting bam file.
samtools index with default parameters for generating corresponding bai file.
For narrow peak calling:
macs2 callpeak -t <test.Bam> -c <input.bam> -f BAM -g 2.9e9 --keep-dup 1 -n <fileNamePrefix> --verbose 3 --outdir <dirName> -B -q 0.05

For broad peak calling:
macs2 callpeak -t <test.bam> -c <input.bam> -f BAM --broad -g 2.9e9 --keep-dup 1 --broad-cutoff 0.05 -n <fileNamePrefix> --verbose 3 --outdir <dirName>

For generating Bigwig file:
bamCompare -b1 <test.bam> -b2 <input.bam> -o <test.bw> -of bigwig --ratio subtract -p <n> --normalizeUsingRPKM --ignoreDuplicates --binSize 1

**Data quality**

FastQC was run on all samples to ensure data quality.
Quality filtering of data was performed using following command:
egrep -w 'NM:i:[0-5]|@SQ|@PG' fileName.sam | awk '($5 == 60 && $2 < 2048) || $1~/^@/' > fileName-Q60.sam.

Number of peaks called with FDR 5% and above 5-fold enrichment:
42D-ENZR-ASCL1-IP: 18,659
42D-ENZR-H3K27ME3-IP: 24,424 (broad)
42D-ENZR-shASCL1-EZH2-IP: 293 (broad)
42D-ENZR-shASCL1-H3K27ME3-IP: 10,336 (broad)
NCI-H660-ASCL1-IP: 36,031

**Software**

FastQC (v0.11.9) for checking raw NGS data quality.
BWA-MEM software (v0.7.17) with default parameters for mapping and alignment of ChIP-seq and ATAC-seq data with reference to hg38 genome.
Samtools software (v1.15) for quality (Q60) filtering and generation of bam files.
MACS2 (v2.2.7.1) for peak calling with FDR q-value 0.05.

deepTools (v3.5.1) for heatmap visualization.
Bedtools (v2.30.0) program suite was used to generate shared and unique peaks.
HOMER (v4.11) for motif analysis.
RUV (v1.26.0) for batch correction of ATAC-seq samples.
DiffBind (v3.2.7) was used for correlation analysis of ATAC-seq samples.
Cufflinks (v. 2.2.1) for RNA-seq.
Gene Set Enrichment Analysis (GSEA, v4.2.2) with pathways from the Molecular Signatures Database (v. 7.1) for pathway analysis.
Single sample GSEA (ssGSEA) was carried out using gProfiler web server

# Flow Cytometry

## Plots

Confirm that:

- [x] The axis labels state the marker and fluorochrome used (e.g. CD4-FITC).
- [x] The axis scales are clearly visible. Include numbers along axes only for bottom left plot of group (a 'group' is an analysis of identical markers).
- [x] All plots are contour plots with outliers or pseudocolor plots.
- [x] A numerical value for number of cells or percentage (with statistics) is provided.

## Methodology

| | |
|---|---|
| Sample preparation | Cells were dissociated using Cellstripper™ (Corning®) at room temperature with gentle shaking and filtered through a 40-µm nylon cell strainer. Single cell suspensions were pelleted at 300 xg and re-suspended in flow cytometry buffer (2mM EDTA, 1% FBS, 0.1% NaN3 in 1x PBS) with fluorophore-conjugated antibodies for 45 min at 4°C. Cells were washed 2x with flow cytometry buffer. |
| Instrument | BD FACSCanto II |
| Software | FACSDiva software (v. 8) was used for cell collection. Data was analyzed using FlowJo software (version 10.4.2). |
| Cell population abundance | Populations studied were composed of cancer cell exclusively and all cells were analyzed. |
| Gating strategy | Forward versus side scatter (FSC vs. SSC) gating was used to identify cells of interest and exclude debris and dead cells. Unstained cells were used as a negative control. Dead cells were excluded using 7-AAD (Thermo cat #A1310). |

- [x] Tick this box to confirm that a figure exemplifying the gating strategy is provided in the Supplementary Information.
